# Methylome-wide association study of multidimensional resilience

**DOI:** 10.1017/S0954579424001330

**Published:** 2024-09-16

**Authors:** Alexandra Y. Vazquez, S. Alexandra Burt, Colter Mitchell, Kelly L. Klump, Luke W. Hyde, Shaunna L. Clark

**Affiliations:** 1 Department of Psychology, Michigan State University, East Lansing, MI, USA,; 2 Institute for Social Research, University of Michigan, Ann Arbor, MI, USA,; 3 Department of Psychology, University of Michigan, Ann Arbor, MI, USA; 4 Department of Psychiatry & Behavioral Sciences, Texas A&M University, College Station, TX, USA

**Keywords:** DNA methylation, epigenetics, neighborhood disadvantage, resilience, Youth

## Abstract

Although resilient youth provide an important model of successful adaptation to adversity, we know relatively little about the origins of their positive outcomes, particularly the role of biological mechanisms. The current study employed a series of methylome-wide association studies to identify methylomic biomarkers of resilience in a unique sample of 276 twins within 141 families residing in disadvantaged neighborhoods. Results revealed methylome-wide significant differentially methylated probes (DMPs) for social and academic resilience and suggestive DMPs for psychological resilience and resilience across domains. Pathway analyses informed our understanding of the biological underpinnings of significant differentially methylated probes. Monozygotic twin difference analyses were then employed to narrow in on DMPs that were specifically environmental in origin. Our findings suggest that alterations in the DNA methylome may be implicated in youth resilience to neighborhood adversity and that some of the suggestive DMPs may be environmentally engendered. Importantly, our ability to replicate our findings in a well-powered sample was hindered by the scarcity of twin samples with youth exposed to moderate to substantial levels of adversity. Thus, although preliminary, the present study is the first to identify DNA methylation biomarkers of academic and social resilience.

Neighborhood disadvantage is a chronic form of adversity that is often characterized by high rates of poverty, limited physical (e.g., green space) and built (e.g., grocery stores, pharmacies) resources, community violence, high exposure to toxicants, and low social cohesion (Jutte et al., 2015; Wodtke et al., 2011). This form of adversity has been demonstrated to have a robust effect on long-term physical (e.g., cardiovascular disease, cancer, obesity) (Cubbin et al., 2006; Jutte et al., 2015) and mental health (e.g., depression, substance use) (Diez Roux & Mair, 2010; Jutte et al., 2015) outcomes. Indeed, neighborhoods have been demonstrated to be a robust predictor of life expectancy discrepancies, future health, and life chances (Evans et al., 2012; Haley et al., 2012; Jutte et al., 2015; Lavizzo-Mourey, n.d.). The effects of neighborhood on health outcomes have also been shown to persist when accounting for individual deprivation and characteristics (Steptoe & Feldman, 2001). Even so, positive adjustment and competent functioning within the context of such adversity or *resilience* (Luthar et al., 2000; Masten, 2001), is quite common (40%–62% of exposed youth) (Luthar et al., 2015; Masten, 2001; Vanderbilt-Adriance & Shaw, 2008). Resilient youth thus provide a model of successful adaptation to adversity as understanding how environmental and biological factors may enable these positive outcomes is of great importance to informing prevention and intervention efforts for youth in disadvantaged neighborhood contexts.

While much of the early literature in the field conceptualized resilience as a static individual trait, contemporary work has explicitly reconceptualized resilience as a dynamic outcome that is influenced by the individual’s attributes, as well as their familial and community-level contexts (Luthar et al., 2000; Masten, 2001; Rutter, 2006). The extant empirical literature on resilience has, in turn, largely focused on socioecological factors, identifying several factors (e.g., parenting behavior) that promote or constrain resilience (Curtis & Cicchetti, 2003). In recent decades, however, a growing number of studies have begun to examine the role of biological mechanisms in the development of resilience (Burt, 2017; Curtis & Cicchetti, 2003; Karatsoreos & McEwen, 2013; Luthar et al., 2000; McEwen et al., 2015; Panter-Brick & Leckman, 2013). Recent theoretical work (e.g., biopsychosocial model) (Feder et al., 2019) specifically highlights the transactional relationship between socioecological and biological influences on youth resilience. One potential mechanism undergirding these transactions relates to epigenetics and the biological embedding of stress via DNA methylation (e.g., silencing or activation of genes). Several epigenetic studies have found evidence of DNA methylation that results from environmental stressors, predicting outcomes ranging from stress response (Smith et al., 2017) to physical health (Notterman & Mitchell, 2015) and depression (Sun et al., 2013).

Given the growing literature examining the role of DNA methylation in response to stressors, it is somewhat surprising to note that the literature examining the role of DNA methylation in resilience to stressors remains scarce. Three published studies have examined DNA methylomic biomarkers of resilience in human samples (Milaniak et al., 2017; Miller et al., 2020), two of which examined DNA methylation in only one or two specific gene regions. Milaniak and colleagues (Milaniak et al., 2017) found that DNA methylation in the oxytocin receptor gene at birth predicted psychological resilience (i.e., a lack of conduct problems) to prenatal environmental stressors in middle childhood (*N* = 321). Similarly, Miller and colleagues (Miller et al., 2020) found that DNA methylation of sites located on the *NR3C1* and *FKBP5* genes predicted psychological resilience (i.e., measured using the Brief Resilience Scale) among emerging to middle-aged adults (*N* = 49). Although these studies begin to provide proof of concept for the idea that DNA methylation is a mechanism supporting resilience to adversity, they were notably limited by their focus on specific gene regions despite the availability of methylome-wide arrays. Indeed, Lu and colleagues (Lu et al., 2023) appear to have conducted the only methylome-wide association study (MWAS) on psychological resilience (*N* = 78; discovery sample *N* = 16, validation sample *N* = 62). While their study identified three differentially methylated probes (DMPs), there are a number of critical limitations of this study. First and foremost, the authors failed to account for multiple testing; given the *p*-values reported, the DMPs they identified would likely not remain statistically significant if appropriate correction methods (e.g., false discovery rate; Benjamini & Hochberg, 1995) were applied. In addition, the study did not control for blood cell-type proportions in their analyses, which is known to lead to inflated test statistics. Also, despite their conceptualization of resilience as adaptation in the context of exposure to substantial stress, adversity, or trauma, the authors do not report whether their sample was restricted to individuals with such exposures (Lu et al., 2023), thus limiting the generalizability of their findings. Of note, all three of these studies are further limited by their focus on only a single form of resilience despite the multidimensional nature of resilience (i.e., individuals may be resilient in one area but not another). Also, Lu et al. (2023) and Miller et al. (2020) employed notably small samples, calling into question the robustness and generalizability of their findings, particularly given that the MWAS conducted by Lu et al. (2023) requires large samples to adequately detect effects. Thus, there is a clear and compelling need for studies to examine DNA methylomic biomarkers of multiple dimensions of resilience (academic, social, psychological, overall) across the entire methylome in sizable samples exposed to moderate to severe levels of adversity.

That said, there are a handful of relevant empirical studies using animal models. For example, Weaver et al., (Weaver et al., 2004), revealed that high levels of maternal care altered DNA methylation at the GR exon 1_7_ promoter site (accompanied by negative effects on the stress response system) (Szyf et al., 2005; Weaver et al., 2004) in the first week of life and persisting into adulthood. What’s more, DNA methylation at this site appeared to be directly programmed by maternal behavior and reversible through the use of a histone deacetylase inhibitor trichostatin A. Elliot and colleagues (Elliott et al.,, 2010) also assessed changes in DNA methylation in rats exposed to a social defeat protocol (Krishnan et al., 2007) and found, that while most mice avoided their neighbor following the protocol, a subset of mice with significantly increased DNA methylation of the *Crf* promoter exhibited behavioral resiliency to the social defeat and interacted with the neighbor. These findings collectively bolster conclusions that both promotive and stressful life events may alter DNA methylation with downstream developmental consequences.

In sum, although research is still limited, there is reason to expect that DNA methylation may be an important component of resilience to adversity. Meaningfully extending this line of work to understand resilience in living humans is trickier than it might seem, however. Although usually discussed as a product of the environment only, DNA methylation is also genetically influenced (Grundberg et al., 2013; Zhang et al., 2014; van Dongen et al., 2016). As such, what may appear to be environmentally induced DNA methylation for a given outcome could in fact reflect genetic effects, a potential confound that undercuts the conclusions of human DNA methylation studies, including the previously discussed MWAS on psychological resilience (Lu et al., 2023). Monozygotic (MZ) twin difference designs are considered the gold standard for overcoming this uncertainty in living humans (Burt et al., 2006). MZ twins are genetically identical and yet can and do have different DNA methylomes as a result of their unique environmental experiences (Fraga et al., 2005). Unfortunately, most twin studies are population-based and include relatively few youths exposed to adversity and even fewer who demonstrate resilience to that adversity. The utilization of a sample enriched for disadvantage to study the role of DNA methylation in resilience would thus offer significant promise for our understanding of differences in adaptability to adversity.

## Current study

The current study aimed to identify DNA methylation biomarkers of resilience in a unique sample of twins enriched for disadvantage. We identified DNA methylation sites associated with academic resilience, social resilience, psychological resilience, and resilience across domains. Analyses were conducted using the entire sample of twins, allowing us to identify general methylomic biomarkers of resilience. We then conducted twin difference analyses of the significant and suggestive CpG sites in only MZ pairs, allowing us to narrow in on those sites that are specifically environmental in origin. We hypothesized that we would find evidence of methylated sites that are associated with resilience (i.e., academic, social, psychological, and across domains) to disadvantage and that differences in DNA methylation between MZ twins will predict differences in their resilience, strengthening causal inferences.

## Methods

### Participants

Participants were recruited as part of the Twin Study of Behavioral and Emotional Development in Children (TBED-C), a study within the population-based Michigan State University Twin Registry (Burt & Klump, 2019). The TBED-C sample encompasses two arms of participants assessed between 2008 and 2015: a population-based arm of 1,054 twins from 528 families recruited from across lower Michigan and an under-resourced arm of 1,000 twins from 502 families residing in modestly to severely disadvantaged neighborhoods in the same recruitment area. Participating twins were screened for cognitive and physical conditions that would impede completion of the assessment (e.g., a significant developmental delay). Children provided informed assent, and informed consent was obtained from parents. Zygosity was determined using physical similarity questionnaires administered to the twins’ primary caregiver (Peeters et al., 1998).

Recruitment procedures are detailed at length in prior work (Burt & Klump, 2019). In brief, families were recruited directly from birth records, or from a population-based registry that was itself recruited via birth records, via anonymous recruitment mailings in conjunction with the Michigan Department of Health and Human Services. Recruitment procedures for the under-resourced sample were restricted to those families residing in neighborhoods where neighborhood poverty was 10.5% (the median for Michigan neighborhoods in 2008) or greater, meaning that 10.5% or more of households were living below the poverty line according to census-level data. The response rate for the population-based and under-resourced arms of the sample was 62% and 57%, respectively. The under-resourced arm of the sample was significantly more racially diverse (15% Black, 75% White) than the population-based arm of the sample, reported lower family income (the means were $72,027 and $57,281, respectively; Cohen’s *d* = −0.38), and had higher paternal felony convictions (*d* = 0.30). The final under-resourced arm of the sample appears representative of the full sample of families we attempted to recruit as indexed via a brief questionnaire administered to approximately 85% of nonparticipating families (Burt & Klump, 2019).

Participants in the current study represent a subsample of the under-resourced arm of the sample, as well a subsample of families from the population-based arm of the sample who would have met the criteria for the under-resourced arm (i.e., those living in neighborhoods with above median poverty). This totaled a possible sample of 768 families residing in disadvantaged neighborhood contexts, of which saliva assays have been completed for 240 twin pairs (the majority of whom were MZ pairs). Following assay quality control procedures and exclusion of participants with insufficient informant data to compute outcomes of interest, 270 participants from 135 full twin pairs (115 MZ; 20 dizygotic [DZ]) and six singletons (i.e., twins without a pair) formed the primary analytic sample for the current study (total *N* = 276 individuals). All 20 DZ pairs were male-male, whereas among MZ pairs, 69 were male-male, and 46 were female-female. The remaining singletons included five males and one female. All twins ranged in age from 6 to 11 years old at the time their questionnaires, and saliva samples were collected. The majority of participants in our final analytic sample identified as White (77.8%), 10.6% identified as Black, 2.1% as Native American, 2.1% as Pacific Islander, 1.4% as Latinx, and 6% identified as “Other” or a race prominent in less than 1% of the sample (i.e., Asian). Finally, the mean level of neighborhood poverty was 23%, while the mean family income was approximately $40,000 for a family of four.

### Measures

As resilience is inherently a conditional construct – in that youth cannot demonstrate resilience without having first been exposed to adversity – it must be inferred through demonstrated competency and positive mental health despite exposure to adversity. In our case, we focused on resilience to moderate to severe neighborhood disadvantage, a form of chronic adversity. Competency and mental health were assessed via maternal reports on the Child Behavior Checklist (CBCL) (Achenbach & Rescorla, 2001). The CBCL is one of the most commonly used and well-validated instruments for assessing academic and social competence, as well as mental health (internalizing and externalizing) problems prior to adulthood (Nakamura et al., 2009).

### Academic resilience

The School Competency subscale of the CBCL served as our continuous measure of academic resilience (*α* = .64). This subscale includes items that assess school performance across subject domains, special education services received, repeated classes, and academic or other school-related problems (e.g., Does your child receive special education or remedial services or attend a special class or special school?). Mothers responded to a four-part question about academic performance on a 4-point scale ranging from “failing” to “above average,” as well as 3 binary (yes/no) questions. Of note, this score was kurtotic due to the narrow range of the subscale and was thus transformed by taking the natural log of each score to remove kurtosis prior to analyses.

### Social resilience

The Social Competency subscale of the CBCL served as our continuous measure of social resilience (*α* = .49). Mothers responded to six questions assessing the child’s involvement in organizations, number of friends, contact with friends, behavior with others, and behavior alone (e.g., About how many times a week does your child do things with any friends outside of regular school hours?). Of note, the lower reliability evidenced in the school and social competence subscales is not uncommon given that they are multidimensional in nature such that most items capture different aspects of social and school competence.

### Psychological resilience

An absence of psychopathology count variable served as our measure of psychological resilience (*α* = .78). Mothers rated the extent to which a series of statements described their child’s behavior during the past 6 months; responses were made on a 3-point scale ranging from 0 (*never*) to 2 (*often/mostly true*). We examined all eight psychopathology scales in the CBCL: anxious/depressed (e.g., fears certain animals, situations, or places, other than school), withdrawn/depressed (e.g., there is very little he/she enjoys), somatic complaints (e.g., constipated, doesn’t move bowels), social problems (e.g., complains of loneliness), thought problems (e.g., hears sounds or voices that aren’t there), attention problems (e.g., can’t concentrate, can’t pay attention for long), rule-breaking (e.g., breaks rules at home, school, or elsewhere), and aggressive behavior (e.g., destroys things belonging to his/her family or others). For the current study, we recoded each of these eight subscales as binary variables that indicate whether the child was at or *above* (0) or *below* (1) the CBCL’s empirically established borderline clinical significance cut point for that scale (Achenbach & Rescorla, 2001). The eight dichotomous variables were then summed to form an absence of psychopathology score ranging from 0 to 8, where a higher score reflects less psychopathology and greater psychological resilience. Of note, this score was negatively skewed due to lower levels of psychopathology in our nonclinical sample and was thus transformed by taking the natural log of each score to reduce the skew prior to analyses.

### Resilience across domains

Consistent with state-of-the-science studies of socio-emotional resilience, we are defining overarching resilience in the face of disadvantage as both the *absence* of psychopathology and the *presence* of social and academic competencies (Luthar et al., 2000; Masten, 2001; Rutter, 2006). Therefore, a dichotomous indicator of resilience across domains was computed with individuals above the CBCL social and academic competency subscale cut points (*t*-score = 40) (Achenbach & Rescorla, 2001) and below the CBCL internalizing and externalizing score borderline cut points (*t*-score = 60) (Achenbach & Rescorla, 2001) considered “resilient” (*N* = 135), whereas all others were considered “non-resilient” (*N* = 141) in at least one domain. Seventy-five twin pairs were concordant for resilience across domains, while 60 pairs were discordant for resilience across domains.

### Assaying the methylome

Saliva samples were collected during the twin family’s assessment using Oragene collection kits (DNA Genotek). DNA was extracted using the Oragene Laboratory Protocol Manual Purification of DNA. Extracted DNA was then sodium bisulfite converted, and methylation was assessed in the converted DNA using the Infinium Human Methylation EPIC Bead Chip (Illumina). DNA conversion and methylation measurement were performed by the University of Michigan Sequencing Core.

Thorough quality control and intra-sample normalization procedures were employed using the Chip Analysis Methylation Pipeline for Illumina HumanMethylation450 and EPIC (ChAMP) Bioconductor package (Butcher & Beck, 2015; Morris et al., 2014). Samples with a high proportion of failed probes (≥10%) were removed (*n* = 1). Probes were removed if their detection *p*-value was above 0.01 (*n* = 86,415 probes), if the bead count was less than 3 in at least 5% of samples (*n* = 3608 probes), if probes aligned to multiple locations (cross-hybridizing probes) (Nordlund et al., 2013), if probes were not located at CpG sites (*n* = 2242), if probes overlapped with single nucleotide polymorphisms, or if probes were located on sex chromosomes (*n* = 12,610). In order to correct for probe design bias, we used the champ.norm function (Teschendorff et al., 2013) of the ChAMP package. The ComBat function of the Surrogate Variable Analysis Bioconductor package was then used to correct for batch effects by slide and then array (Leek, 2020). Finally, cell-type proportions were estimated for the most common cell types in saliva using the Epigenetic Dissection of Intra-Sample-Heterogeneity (EpiDISH) Bioconductor package (Zheng et al., 2018). These procedures yielded DNA methylation values (log2 methylated/unmethylated DNA at a specific probe, i.e., *M*-values) across 728,396 CpG sites for 276 participants.

### Methylome-wide association study (MWAS)

The MWAS was performed on the full sample (*N* = 276) using regression to identify DNA methylation sites that were associated with resilience (i.e., social, academic, psychological, and across domains), so-called DMPs. Specifically, we fit logistic and ordinary least squares regression models in R for our dichotomous (i.e., resilience across domains) and continuous/discrete (i.e., social, academic, and psychological resilience) outcomes, respectively. To account for the nonindependence of twins within pairs, we corrected the standard errors by fitting our models within a heteroskedasticity-consistent covariance matrix estimator using the sandwich package in R (Zeileis, 2006). To control for potential confounders, we included sex, age, zygosity, ethnicity, and estimated cell-type proportions as covariates in our models. A *p*-value threshold of *P* < 9 × 10^−8^ was used to declare a DMP methylome-wide significant (Mansell et al., 2019) and *P* < 1 × 10^−5^ for suggestive DMPs (Lander & Kruglyak, 1995).

### Pathway analysis

To gain insight into the biological pathways affected by resilience, we used ConsensusPathDB (Kamburov et al., 2009, 2011) to test for overrepresentation of top suggestive MWAS findings located within genes in the biological pathways in the Reactome (Croft et al., 2014) database. For a pathway to be considered enriched, a cut point of *P* < 0.01 was utilized, and at least two genes among the top MWAS findings had to be present in the pathway.

### Monozygotic twin difference analyses

Finally, we performed twin difference analyses in R in which we compared MZ co-twins to strengthen causal inferences. Because MZ co-twins cannot differ in their epigenome as a consequence of genetic differences (as they are genetically identical), any differences in the methylome of co-twins point toward environmental mediation. We computed differences in DNA methylation scores for the significant and suggestive DMPs from the MWASs as well as for the four resilience phenotypes. For our twin difference analyses, we regressed DNA methylation difference scores for the DMPs and covariates (i.e., sex, age, and ethnicity, each on the twin-pair level) on resilience (i.e., academic, social, psychological, and across domains) difference scores. DMPs were then compared to a 95% statistical significance threshold (*p* ≤ 0.05).

## Results

### Descriptive statistics

Descriptive statistics for resilience across domains, psychological resilience, academic resilience, and social resilience are available in Table 1. While scores for psychological, academic, and social resilience were continuous, the score for general resilience across domains was dichotomous. Approximately half of the participants were considered to be resilient across domains. The majority of participants exhibited high scores for psychological and academic resilience; however, social resilience scores were more variable. Finally, the means and standard deviations of the four resilience phenotypes in MZ twins and DZ twins were equivalent. Pearson correlations between all cell type proportion estimates and the four resilience phenotypes were first examined; none were significant (Table S1).

A large proportion of co-twins differed in their degree of resilience, as indexed dimensionally (Table 1). Most co-twins (71%) had different levels of social resilience, with a mean co-twin difference of 43% of the typical phenotypic standard deviation across the full sample. Roughly half of the co-twins (45%) had different levels of academic resilience, with a mean co-twin difference of 38% of the typical phenotypic standard deviation across the full sample. Finally, although only a third (36%) of co-twins evidenced different levels of psychological resilience, those that differed did so quite a bit, with a mean co-twin difference that was 59% of the typical phenotypic standard deviation across the sample. For our dichotomous phenotype of resilience across domains, 46% of co-twins were discordant.

As a final point, we note that no co-twins across the entire sample had identical DNAm scores for any of the 728,396 CpG sites. The observed differences were quite large. The mean co-twin difference was 300% of the typical DNAm standard deviation across the full sample. What’s more, even when concordance was evaluated somewhat liberally (i.e., a co-twin difference of .001 or less; DNAm range: 0–1), twin pairs remained discordant on 94–98% of the CpG sites. When discordance was evaluated quite liberally (i.e., a co-twin difference of .01 or less), twin pairs were still discordant on 52–80% of the CpG sites.

### Methylome-wide association study (MWAS)

**Figure 1.**
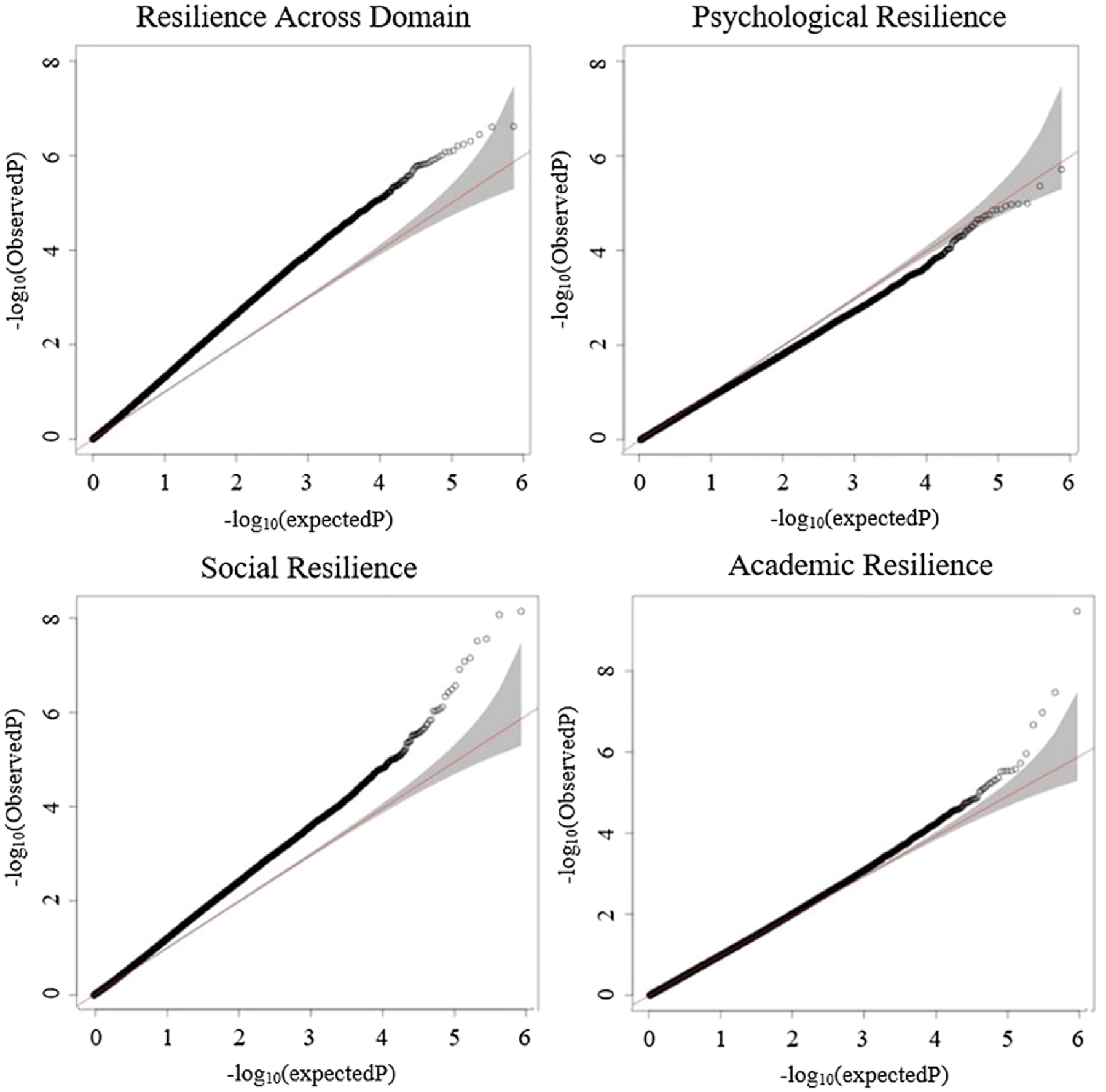
Quantile-quantile plots for MWAS of each resilience domain. *Note*. The observed *p-*values (black open circles), on a -log10 scale, are plotted against their expected values (red main diagonal line) under the null hypothesis assuming none of the CpGs have an effect. Shaded grey bands indicate the 95% confidence bands (CI).

The quantile-quantile plots for each of the resilience outcomes are shown in Figure 1. The number of points above the 95% confidence interval, deviating from the line of expected points according to the null hypothesis, indicates a considerable number of statistically significant or suggestive findings for resilience across domains, academic resilience, and social resilience. However, the plot for psychological resilience does not depict points above the 95% confidence interval, suggesting limited significant results for this outcome.

The top 10 significant (*P* < 9 × 10^−8^; Mansell et al., 2019) and/or suggestive (*P* < 1× 10^−5^; Lander & Kruglyak, 1995) MWAS DMPs and test statistics for each outcome are provided in Table 2 with covariate results for these DMPs provided in Table S2 and full results available in Table S3. Results indicated that, although there were no methylome-wide significant DMPs associated with resilience across domains, there were 90 suggestive DMPs. One of the top suggestive DMPs was located in an intron of *SOX30*, which is a member of the SOX family of transcription factors involved in determining cell fate and regulating embryonic development (Osaki et al., 1999).

The psychological resilience MWAS yielded no methylome-wide DMPs, but two suggestive ones. The top suggestive DMP was located in an intron of *HOXC13*, which has been implicated in cancer prognosis and belongs to the homeobox family of genes that encode transcription factors involved in morphogenesis (Panagopoulos et al., 2003).

There were two methylome-wide significant and 20 suggestive DMPs associated with academic resilience. The top methylome-wide significant DMP was located in an intron of *MYO10*, which encodes a member of the myosin superfamily proteins and is associated with an increased risk for childhood apraxia of speech (Peter et al., 2016). A top suggestive DMP was located in an intron of *BRF1*, which encodes a subunit of the RNA polymerase III transcription initiation factor and has been associated with neurodevelopmental abnormalities (Borck et al., 2015).

Finally, there were six methylome-wide significant and 54 suggestive DMPs associated with social resilience. The top methylome-wide significant DMP was located in an intron and CpG island of *AC006372.5*, also known as *LOC101927914*, an uncharacterized RNA gene. The second top methylome-wide significant DMP, as well as a suggestive DMP, was located in an intron of *HLA-DRB1*. In addition, another suggestive DMP was located in an intron of *HLA-DQB2*. *HLA-DRB1* and *HLA-DQB2* are located in the HLA region on chromosome 6, a large region of linkage disequilibrium indicating that these may not be independent signals (Simmonds & Gough, 2007).

Sensitivity analyses were also performed on the normalized data with slide and array entered as covariates to verify that the ComBat correction we employed was not inflating results (Zindler et al., 2020); this alternative approach yielded a greater number of significant and suggestive probes, suggesting that our approach was more conservative.

### Enriched pathways

The majority of significant or suggestive DMPs were located in unique genes; 76 of 90 for resilience across domains, 2 of 2 for psychological resilience, 16 of 22 for academic resilience, and 47 of 60 for social resilience. All of the significantly enriched pathways are provided in Table 3. Resilience across domains yielded four significantly enriched pathways. The top significant pathway was the “Listeria Monocytogenes Entry into Host Cells,” which is involved in regulating the entry of bacterium that cause the majority of foodborne outbreaks. No prominent theme emerged among these results. There were no significant enriched pathways for psychological resilience, likely due to the small number of significant or suggestive DMPs for this outcome.

For academic resilience, we observed eight significantly enriched pathways. The *POLR2L* and *BRF1* genes were found in five pathways implicated in the transcription or initiation of RNA polymerase III. RNA polymerase III serves as a catalyst for the synthesis of small RNAs (e.g., *tRNAs, 5S rRNA, snRNA*) considered to be essential for various cellular functions (Abascal-Palacios et al., 2018). The *POLR2L* gene encodes a subunit of RNA polymerase I, II, and III and is therefore heavily involved in synthesizing messenger RNAs (Acker et al., 1996). In addition, the *POLR2L* and LI*G3* genes were found in three pathways involved in gap-filling and nucleotide excision DNA repairs. As a member of the DNA ligase family, the *LIG3* gene is involved in excision repairs and has been linked to increased risk for cancer (Li et al., 2009; Li et al., 2018), neural tube defects (Li et al., 2018), Alzheimer disease (Kwiatkowski et al., 2016), and recurrent depression (Czarny et al., 2017).

Social resilience evidenced nine significantly enriched pathways. The *HLA-DRB1* and *HLA-DQB2* genes appeared in eight of these pathways, most of which are involved in T-cell receptor signaling, indicating that these results were driven by the HLA region on chromosome 6. The HLA region includes several genes – such as the *HLA-DRB1* and *HLA-DQB2* genes – that play a central role in immune system functioning (Simmonds & Gough, 2007). The HLA region is associated with longevity (Joshi et al., 2017), cognitive ability (Payton et al., 2006), and mental health disorders (e.g., schizophrenia, autism) (Bennabi et al., 2018; Halley et al., 2013).

### Monozygotic twin differences

For our final analyses, we sought to evaluate the extent to which the significant and suggestive DMPs from each of the MWAS models above were environmental in origin via MZ twin differences analyses. Results are provided in Table 4. Two DMPs for resilience across domains differed significantly across MZ pairs. The top DMP was located in *Y_RNA*, a class of small non-encoding RNAs involved in the repression of Ro60 (i.e., a protein that has been implicated in responses to environmental stress) as well as the initiation of chromosomal DNA replication (Christov et al., 2006). The second top DMP was located in an intron of *TMEM67*, a gene needed to facilitate ciliary structure and function (Yinsheng et al., 2022); defects can cause Joubert syndrome (characterized by abnormal brain development) and Meckel syndrome (most commonly characterized by enlarged kidneys).

Four DMPs for social resilience also differed significantly across MZ pairs. The top DMP was located in an intron of *LINC01250*, a long intergenic non-protein coding RNA gene (Dungan et al., 2021). The second top DMP was located in an intron of *PIGG*, a protein-coding gene involved in glycosylphosphatidylinositol-anchor biosynthesis; allelic variants of *PIGG* have been linked to intellectual disability with hypotonia and seizures (Makrythanasis et al., 2016).

For academic resilience, two DMPs differed significantly across MZ pairs. The top DMP was located in an intron of *RABEP2*, a protein-coding gene that enables GTPase activator activity and growth factor activity (Kofler et al., 2018). The second top DMP was located in an intron and CpG Island of the aforementioned BRF1. DMPs for psychological resilience did not differ across MZ pairs.

## Discussion

The goal of this study was to identify epigenetic correlates of resilience to neighborhood disadvantage in a sample of living humans. MWAS analyses conducted in 276 twins within 141 families revealed a handful of methylome-wide significant DMPs associated with academic as well as social resilience, and suggestive DMPs associated with each of the four resilience phenotypes examined (i.e., psychological, academic, social, and across domains). Pathway analyses revealed significantly enriched pathways for academic and social resilience, as well as resilience across domains. Results for academic resilience to neighborhood disadvantage pointed to DNA methylation in pathways related to DNA repair as well as the transcription and initiation of RNA polymerase III. DNA damage typically triggers a response that includes DNA repair. Dysregulation of DNA damage responses can result in developmental and neurological defects (Lee et al., 2016). As mentioned previously, RNA polymerase III is involved in transcribing small RNAs. Dysregulation of small RNAs is thought to be implicated in abnormal brain development (Chang et al., 2009). Taken together, these findings suggest that DNA methylation in these two pathways may alter or inhibit the regulation of DNA damage responses and small RNAs.

These enriched pathways also highlight the role of DNA methylation of the *BRF1* gene in academic resilience. Mutations in *BRF1* have been shown to cause central nervous system and neurodevelopmental anomalies due to a reduction in protein activity. It has been suggested that RNA polymerase III transcription initiated by *BRF1* is necessary for typical cognitive development (Borck et al., 2015), a process that may be affected by DNA methylation of *BRF1*. The current study extends this line of work by demonstrating that an increase in DNA methylation of *BRF1* is associated with academic resilience, a construct that is thought to be correlated with cognitive ability (Mayes et al., 2009; Tiet et al., 1998).

Results also suggest that DNA methylation in genes located in the HLA region involved in T-cell receptor (TCR) signaling may play a role in social resilience to neighborhood disadvantage. TCR signaling refers to cellular signaling cascades involved in determining cell fate, including cell survival, differentiation, and proliferation. TCRs typically bind to proteins involved in the immune response. Recent studies have demonstrated that proteins involved in the immune response are expressed in the central nervous system and play critical roles in synaptic transmission and plasticity, as well as refinement of connections during brain development (Garay & McAllister, 2010). Thus, DNA methylation of genes involved in TCR signaling may have downstream effects on brain development. Research on social cognition has demonstrated that the portions of the temporal lobe, the amygdala, and the cingulate cortex are implicated in social behavior via their involvement in the perception of social stimuli and the ability to link these stimuli to emotion, motivation, and cognition (Adolphs, 2001). Therefore, while additional research is needed to confirm that TCR signaling impacts these brain regions in particular, this may explain its relationship with interpersonal functioning and social resilience (Cook et al., 1994).

MZ twin difference analyses revealed two suggestive DMPs for resilience across domains, two for academic resilience, and four for social resilience. While none of the significant methylome-wide DMPs differed across MZ twins, the suggestive DMPs that did differ across MZ pairs were located in genes implicated in responses to environmental stress and neurodevelopmental abnormalities.

Since MZ twins are genetically identical, significant findings point toward environmentally engendered DNA methylation in those cases. Alternatively, the absence of significant MZ differences in our methylome-wide significant DMPs suggests that those DMPs may not reflect causal environmental processes per se. This suggests that while some DMPs appear to be environmental in origin, others point to the possibility of genetic or developmental mediation of those methylomic effects. However, null results may also reflect family-wide influences or MZ differences that were too small to capture environmental mediation.

### Limitations

The unique twin design of this study coupled with the relatively high degree of disadvantage experienced by participants uniquely positioned us to detect DMPs for resilience that are environmental in origin. However, there are limitations of the current study that are important to consider. First, because DNA methylation can be tissue-specific, etiological interpretations of saliva-based DNA methylation must be made with caution, the minimum interpretation being that DMPs could potentially be biomarkers of resilience. Given that our study did not contain a replication sample, our results are provisional and warrant further investigation with an independent sample. That being said, we are not aware of a second child twin sample experiencing sufficiently high rates of adversity to study resilience to that adversity at this time. Thus, diverse twin samples with higher rates of adverse exposures are needed to facilitate future replication. Next, although our sample is representative of racial and ethnic demographics throughout the state of Michigan, the racial breakdown of the sample is still primarily White, thereby limiting the generalizability of our findings to racially minoritized communities. It would be critical for future methylomic studies of resilience to recruit racially diverse samples. Lastly, while this study focuses specifically on resilience to neighborhood disadvantage, other forms of resilience may have distinct methylomic markers (e.g., resilience to maltreatment). Additional research on other forms of resilience would facilitate a comparison of methylomic markers across distinct forms of resilience.

### Implications

Overall, this is one of the first studies to uncover potential DNA methylomic biomarkers of resilience in a sample of living humans. Our findings preliminarily highlight DNA methylation as a potential biological mechanism implicated in resilient outcomes, in that we identified a handful of methylome-wide significant and suggestive DNA methylation sites that predict resilience to neighborhood disadvantage. The etiologic inferences we can make about these DMPs and genes are more limited, however, since the significant DMPs from the MWAS did not differ across MZ twins. Such results support the possibility of genetic or developmental mediation for those DMPs. That said, we did identify a handful of suggestive methylomic correlates of resilience that differed across MZ twins. These environmental changes in the methylome are also at least nominally consistent with the biopsychosocial model’s theory in that they point to the importance of environmental effects, as well as reciprocal feedback between biology and the environment. Although beyond the scope of the current study, we intend to expand on our findings by examining the effect of specific environmental promotive factors (e.g., parental warmth) on DNAm sites implicated in youth resilience in the near future.

## Supplementary Material

Vazquez supplement

**Supplementary material.** The supplementary material for this article can be found at https://doi.org/10.1017/S0954579424001330.

## Figures and Tables

**Table 1.** Descriptive statistics

Construct	Monozygotic Twins (MZ)								Dizygotic Twins (DZ)				
	Mean	SD	Min	Max	*N*	Disc.	Tw diff. mean	Tw diff. SD	Mean	SD	Min	Max	*N*
**Resilience across domains**	0.50	.50	.00	1.00	240	45.7%	–	–	0.39	.49	.00	1.00	41
**Psychological resilience**	5.52	.93	.00	6.00	237	36.2%	0.55	0.92	5.51	.98	2.00	6.00	41
**Academic resilience**	4.85	1.12	.00	6.00	238	44.9%	0.43	0.65	4.54	1.17	1.50	6.00	40
**Social resilience**	7.42	2.26	1.00	13.50	238	69.7%	0.99	1.05	7.12	2.63	2.50	13.50	41
**DNAm**	0.59	0.01	0	1	230	100%	.03	.01	0.59	.01	0	1	40

*Note*. On the left are the descriptive statistics across individuals who are in a monozygotic twin pair, and on the right are the descriptive statistics across individuals who are in a dizygotic twin pair. Means, standard deviations (SD), minimums (Min), maximums (Max), and sample size (N) are presented for each of the four resilience phenotypes and DNA methylation estimates (DNAm). The proportion of discordant co-twins (disc.; i.e., nonidentical scores), co-twin mean difference scores (Tw diff. mean), and the standard deviation for co-twin mean difference scores (Tw diff. SD) are also presented for monozygotic twins.

**Table 2.** Top 10 significant and/or suggestive differentially methylated probes

Model	Probe	Chr	Start	Beta	*Z*/*T*-value	*P*-value	Gene	Genomic features
Resilience across domains	cg08862567	20	33447234	80.275	5.161	2.452E - 07	*GGT7*	Intron; CpG island
	cg15869383	19	58258088	−129.038	− 5.087	3.630E - 07	*ZNF776*	Intron; CpG island
	cg23044017	19	36822441	−79.445	− 5.026	5.013E - 07	*LINC00665*	Exon; CpG island
	cg02536150	10	17754084	45.363	4.981	6.314E - 07	*STAM*	Intron
	cg24059404	4	184580365	−193.388	− 4.937	7.929E - 07	*RWDD4*	Exon
	cg24221965	15	81422778	23.580	4.925	8.436E - 07	*C15orf26*	Intron
	cg16373426	5	157079899	88.290	4.924	8.499E - 07	*SOX30*	Intron
	cg09114799	12	48152514	−242.566	− 4.881	1.056E - 06	*RAPGEF3*	Exon
	cg18056754	11	122955452	62.652	4.860	1.172E - 06	*CLMP*	Intron
	cg03078854	6	32810000	96.825	4.850	1.233E - 06	*PSMB8*	Exon
Psychological resilience	cg00059246	12	54337928	3.673	4.866	1.957E - 06	*HOXC13*	Intron
	cg10674017	2	3201975	−15.245	− 4.689	4.405E - 06	*TSSC1*	Intron
Academic resilience	cg09169455	5	16843339	−2.185	− 6.528	3.399E - 10	*MYO10*	Intron
	cg27413290	8	144552724	−4.250	− 5.687	3.422E - 08	*ZC3H3*	Intron; CpG island
	cg23901896	1	201976445	−10.226	− 5.465	1.073E - 07	*ELF3*	Intron
	cg22018084	2	69038737	−2.543	− 4.874	1.887E - 06	*ARHGAP25*	Intron
	cg03116740	11	841334	3.376	4.799	2.668E - 06	*POLR2L*	Intron
	cg20678377	20	47667339	−2.715	− 4.780	2.909E - 06	*CSE1L*	Intron
	cg09895822	14	105738159	8.444	4.778	2.947E - 06	*BRF1*	Intron; CpG island
	cg16444294	16	28925789	17.201	4.773	3.004E - 06	*RABEP2*	Exon
	cg00421032	4	22493280	9.058	4.772	3.025E - 06	*GPR125*	Intron
	cg08857221	1	37941360	4.155	4.694	4.315E - 06	*ZC3H12A*	Exon
Social resilience	cg22321318	7	157294387	17.100	5.979	7.231E - 09	*AC006372.5*	Intron; CpG island
	cg17416722	6	32554384	6.440	5.728	2.753E - 08	*HLA* - *DRB1*	Intron
	cg25960393	8	9106558	5.018	5.708	3.064E - 08	*RP11 – 115J16.1*	Exon
Social resilience	cg14321269	17	6658197	17.674	5.546	7.061E - 08	*XAF1*	Exon
	cg25998860	5	126853953	−114.782	−5.512	8.389E - 08	*PRRC1*	Intron
	cg15559076	11	128109596	18.105	5.439	1.220E - 07	*RP11 – 702B10.1*	Intron
	cg11070274	8	9106609	5.106	5.278	2.721E - 07	*RP11 – 115J16.1*	Exon
	cg20424973	2	3045240	40.116	5.209	3.811E - 07	*LINC01250*	Intron
	cg10985094	17	3631481	23.115	5.064	7.701E - 07	*ITGAE*	Intron
	cg12738264	7	148725794	−210.602	−5.044	8.463E - 07	*PDIA4*	Exon; CpG island

*Note*. ‘Probe’ is the name of the CpG probe in the human reference genome hg19/GRCh37, ‘Chr’ is chromosome, ‘start’ is the base pair location of the probe, ‘gene’ is the gene the probe is located in, and ‘genomic feature’ indicates if the probe is located in an intron, exon, or CpG island. Also shown are the signed test statistic values for regression: ‘*Z*-value’ for the dichotomous outcome of resilience across domains, ‘*T*-value’ for the continuous outcomes, ‘*P*-values,’ and ‘beta’ or regression coefficient. The top 10 methylome-wide significant (*P* - value ≤ 9 × 10^−8^) and/or suggestive (*P* - value ≤ 1 × 10^−5^) MWAS DMPs are displayed for each outcome.

**Table 3.** Enriched pathways

Model	Pathway	*p*-value	*q*-value	Effective size	Gene overlap
Resilience across domains	Listeria monocytogenes entry into host cells	0.002	0.085	19	*CTNNB1; STAM*
	BBSome-mediated cargo-targeting to cilium	0.003	0.085	23	*BBS7; LZTFL1*
	Endosomal sorting complex required for transport	0.005	0.109	32	*STAM; VPS37C*
	Organelle biogenesis and maintenance	0.009	0.126	240	*PRKAG1; TMEM67; BBS7; LZTFL1*
Academic resilience	RNA polymerase III transcription initiation from Type 2 promoter	0.000	0.002	27	*POLR2L; BRF1*
	RNA polymerase III transcription initiation from Type 1 promoter	0.000	0.002	28	*POLR2L; BRF1*
	RNA polymerase III transcription initiation	0.000	0.002	36	*POLR2L; BRF1*
	RNA polymerase III abortive and retractive initiation	0.001	0.002	41	*POLR2L; BRF1*
	RNA polymerase III transcription	0.001	0.002	41	*POLR2L; BRF1*
	Gap-filling DNA repair synthesis and ligation in TC-NER	0.002	0.003	68	*POLR2L; LIG3*
	Transcription-coupled nucleotide excision repair (TC-NER)	0.002	0.004	81	*POLR2L; LIG3*
	Nucleotide excision repair	0.005	0.007	113	*POLR2L; LIG3*
Social resilience	Phosphorylation of CD3 and TCR zeta chains	0.000	0.002	30	*HLA-DRB1; PTPRJ; HLA-DQB2*
	TCR signaling	0.000	0.016	72	*HLA-DRB1; PTPRJ; HLA-DQB2*
	Translocation of ZAP-70 to immunological synapse	0.002	0.033	27	*HLA-DRB1; HLA-DQB2*
	PD-1 signaling	0.002	0.033	31	*HLA-DRB1; HLA-DQB2*
	Generation of second messenger molecules	0.003	0.039	41	*HLA-DRB1; HLA-DQB2*
	Interferon signaling	0.005	0.039	158	*XAF1; HLA-DRB1; HLA-DQB2*
	Downstream TCR signaling	0.005	0.039	51	*HLA-DRB1; HLA-DQB2*
	Neurexins and neuroligins	0.007	0.039	57	*SYT9; SYT1*
	MHC class II antigen presentation	0.007	0.039	59	*HLA-DRB1; HLA-DQB2*

*Note*. ‘Pathway’ is the name of the significantly enriched pathway from the Reactome database, ‘effective size’ is the number of genes involved in the corresponding pathway, and ‘gene overlap’ provides the names of genes from the MWAS that are present in the pathway. Also shown are the signed test statistic values for the pathway analyses: ‘*p*-value’ and ‘*q*-value.’

**Table 4.** Significant monozygotic twin difference differentially methylated probes

Model	Probe	Chr	Start	Beta	*Z*/*T*-value	*P-* value	Gene	Genomic features	Gender beta (SE)	Age beta (SE)	Race beta (SE)
Resilience across domains	Cg10426797	17	7169573	− 27.276	− 2.907	0.004	*Y_RNA*		0.125 (.134)	− 0.048 (.046)	0.168 (.165)
	Cg22687346	8	94767371	49.596	2.407	0.018	*TMEM67*	Intron	0.119 (.135)	− 0.043 (.047)	0.087 (.170)
Academic resilience	Cg09895822	14	105738159	7.392	2.599	0.011	*BRF1*	Intron; CpG island	− 0.020 (.069)	0.011 (.024)	0.095 (.085)
	Cgl6444294	16	28925789	16.136	2.976	0.004	*RABEP2*	Intron	− 0.036 (.069)	0.007 (.023)	0.082 (.084)
Social resilience	Cg09826506	4	522635	− 21.038	− 2.226	0.028	*PIGG*	Intron	0.129 (.285)	− 0.091 (.097)	0.182 (.350)
	Cgl3988209	11	69683042	−11.164	− 2.186	0.031			0.201 (.290)	− 0.128 (.097)	0.289 (.354)
	Cg20140488	22	25463865	− 6.462	− 2.336	0.021	*KIAA1671*	Intron	0.118 (.284)	− 0.119 (.097)	0.187 (.349)
	Cg20424973	2	3045240	−19.613	− 2.009	0.047	*LINC01250*	Intron	0.065 (.285)	− 0.122 (.098)	0.093 (.353)

*Note*. ‘Probe’ is the name of the probe in the human reference genome hg19/GRCh37, ‘Chr’ is chromosome, ‘start’ is the base pair location of the probe, ‘gene’ is the gene the probe is located in, and ‘genomic feature’ indicates if the probe overlaps with introns, exons, or CpG islands. Also shown are the signed test statistic values for regression: ‘*Z*-value’ for the dichotomous outcome of resilience across domains, ‘*T*-value’ for the continuous outcomes, ‘*P*-values,’ and ‘eta’ or regression coefficient. Finally, beta values for all covariates (i.e., ‘gender beta,’ ‘age beta,’ and ‘race beta’) are provided; no covariates’ beta values were significant at *p* < .05. All significant (P ≤ .05) DMPs are provided for each of the outcomes.

## References

[R1] Abascal-Palacios G, Ramsay EP, Beuron F, Morris E, & Vannini A (2018). Structural basis of RNA polymerase III transcription initiation. Nature, 553(7688), 301–306. 10.1038/nature25441 29345637

[R2] Achenbach TM, & Rescorla LA (2001). Manual for the ASEBA school-age forms & profiles: Child behavior checklist for ages 6–18, teacher’s report form, youth self-report: an integrated system of multi-informant assessment. University of Vermont, Research Center for Children Youth & Families.

[R3] Acker J, Murroni O, Mattei M-G, Kedinger C, & Vigneron M(1996). The gene (POLR2L) encoding the hRPB7.6 subunit of human RNA polymerase. Genomics, 32(1), 86–90. 10.1006/geno.1996.0079 8786124

[R4] Adolphs R (2001). The neurobiology of social cognition. Current Opinion in Neurobiology, 11(2), 231–239. 10.1016/S0959-4388(00)00202-6 11301245

[R5] Benjamini Y, & Hochberg Y (1995). Controlling the false discovery rate: A practical and powerful approach to multiple testing. Journal of the Royal Statistical Society: Series B (Methodological), 57(1), 289–300. 10.1111/j.2517-6161.1995.tb02031.x

[R6] Bennabi M, Gaman A, Delorme R, Boukouaci W, Manier Céline, Scheid, Mohammed N, Bengoufa D, Charron D, Krishnamoorthy R, Leboyer M, & Tamouza R (2018). HLA-class II haplotypes and autism spectrum disorders. Scientific Reports, 8(1), 7639. 10.1038/s41598-018-25974-9 29769579 PMC5955937

[R7] Borck G, Hög F, Dentici ML, Tan PL, Sowada N, Medeira A, Gueneau L, Thiele H, Kousi M, Lepri F, Wenzeck L, Blumenthal I, Radicioni A, Schwarzenberg TL, Mandriani B, Fischetto R, Morris-Rosendahl DJ, Altmüller J, Reymond A, Nürnberg P, Merla G, Dallapiccola B, Katsanis N, Cramer P, & Kubisch C (2015). BRF1 mutations alter RNA polymerase III-dependent transcription and cause neurodevelopmental anomalies. Genome Research, 25(2), 155–166. 10.1101/gr.176925.114 25561519 PMC4315290

[R8] Burt SA (2017). Editorial: Finding the silver lining: Incorporating resilience and adaptiveness into studies of psychopathology. Journal of Child Psychology and Psychiatry, 58(5), 529–531. 10.1111/jcpp.12732 28414864

[R9] Burt SA, & Klump KL (2019). The michigan state university twin registry (MSUTR): 15 years of twin and family research. Twin Research and Human Genetics, 22(6), 741–745. 10.1017/thg.2019.57 31466551 PMC7083329

[R10] Burt SA, McGue M, Iacono WG, & Krueger RF (2006). Differential parent-child relationships and adolescent externalizing symptoms: Cross-lagged analyses within a monozygotic twin differences design. Developmental Psychology, 42(6), 1289–1298. 10.1037/0012-1649.42.6.1289 17087561 PMC2365490

[R11] Butcher LM, & Beck S (2015). Probe lasso: A novel method to rope in differentially methylated regions with 450K DNA methylation data. Methods, 72, 21–28. 10.1016/j.ymeth.2014.10.036 25461817 PMC4304833

[R12] Chang S, Wen S, Chen D, & Jin P (2009). Small regulatory RNAs in neurodevelopmental disorders. Human Molecular Genetics, 18(R1), R18–R26. 10.1093/hmg/ddp072 19297398 PMC2657940

[R13] Christov CP, Gardiner TJ, Szüts D, & Krude T (2006). Functional requirement of noncoding Y RNAs for human chromosomal DNA replication. Molecular and Cellular Biology, 26(18), 6993–7004. 10.1128/MCB.01060-06 16943439 PMC1592862

[R14] Cook ET, Greenberg MT, & Kusche CA (1994). The relations between emotional understanding, intellectual functioning, and disruptive behavior problems in elementary-school-aged children. Journal of Abnormal Child Psychology, 22(2), 205–219. 10.1007/BF02167900 8064029

[R15] Croft D, Mundo AF, Haw R, Milacic M, Weiser J, Wu G, Caudy M, Garapati P, Gillespie M, Kamdar MR, Jassal B, Jupe S, Matthews L, May B, Palatnik S, Rothfels K, Shamovsky V, Song H, Williams M, Birney E, Hermjakob H, Stein L, & D’Eustachio P (2014). The reactome pathway knowledgebase. Nucleic Acids Research, 42(D1), D472–D477. 10.1093/nar/gkt1102 24243840 PMC3965010

[R16] Cubbin C, Sundquist K, Ahlén H, Johansson S-E, Winkleby MA, & Sundquist J (2006). Neighborhood deprivation and cardiovascular disease risk factors: Protective and harmful effects. Scandinavian Journal of Public Health, 34(3), 228–237. 16754580 10.1080/14034940500327935

[R17] Curtis WJ, & Cicchetti D (2003). Moving research on resilience into the 21st century: Theoretical and methodological considerations in examining the biological contributors to resilience. Development and Psychopathology, 15(3), 773–810. 10.1017/S0954579403000373 14582940

[R18] Czarny P, Kwiatkowski D, Toma M, Kubiak J, Sliwinska A, Talarowska M, Szemraj J, Maes M, Galecki P, Sliwinski T (2017). Impact of single nucleotide polymorphisms of base excision repair genes on DNA damage and efficiency of DNA repair in recurrent depression disorder. Molecular Neurobiology, 54(6), 4150–4159. 10.1007/s12035-016-9971-6 27324896 PMC5509815

[R19] Diez Roux AV, & Mair C (2010). Neighborhoods and health. Annals of the New York Academy of Sciences, 1186(1), 125–145. 10.1111/j.1749-6632.2009.05333.x 20201871

[R20] Dungan JR, Qin X, Hurdle M, Haynes CS, Hauser ER, & Kraus WE (2021). Genome-wide variants associated with longitudinal survival outcomes among individuals with coronary artery disease. Frontiers in Genetics, 12, 661497. https://www.frontiersin.org/journals/genetics/articles/10.3389/fgene.2021.661497 34140969 10.3389/fgene.2021.661497 PMC8204081

[R21] Elliott E, Ezra-Nevo G, Regev L, Neufeld-Cohen A, & Chen A (2010). Resilience to social stress coincides with functional DNA methylation of the crf gene in adult mice. Nature Neuroscience, 13(11), 1351–1353. 10.1038/nn.2642 20890295

[R22] Evans B, Zimmerman E, Woolf S, & Haley A (2012). Social determinants of health and crime in post-katrina orleans parish.

[R23] Feder A, Fred-Torres S, Southwick SM, & Charney DS (2019). The biology of human resilience: Opportunities for enhancing resilience across the life span. Biological Psychiatry, 86(6), 443–453. 10.1016/j.biopsych.2019.07.012 31466561

[R24] Fraga MF, Ballestar E, Paz MF, Ropero S, Setien F, Ballestar ML, Heine-Suñer D, Cigudosa JC, Urioste M, Benitez J, Boix-Chornet M, Sanchez-Aguilera A, Ling C, Carlsson E, Poulsen P, Vaag A, Stephan Z, Spector TD, Wu Y-Z, Plass C, & Esteller M (2005). Epigenetic differences arise during the lifetime of monozygotic twins. Proceedings of the National Academy of Sciences of the United States of America, 102(30), 10604–10609. 10.1073/pnas.0500398102 16009939 PMC1174919

[R25] Garay P, & McAllister AK (2010). Novel roles for immune molecules in neural development: Implications for neurodevelopmental disorders. Frontiers in Synaptic Neuroscience, 2, 136. https://www.frontiersin.org/articles/10.3389/fnsyn.2010.00136 21423522 10.3389/fnsyn.2010.00136 PMC3059681

[R26] Grundberg E, Meduri E, Sandling JK, Hedman Åsa K., Keildson S, Buil A, Busche S, Yuan W, Nisbet J, Sekowska M, Wilk A, Barrett A, Small KS, Ge B, Caron M, Shin S-Y, Lathrop M, Dermitzakis ET, McCarthy MI, … Zondervan KT (2013). Global analysis of DNA methylation variation in adipose tissue from twins reveals links to disease-associated variants in distal regulatory elements. The American Journal of Human Genetics, 93(5), 876–890. 10.1016/j.ajhg.2013.10.004 24183450 PMC3824131

[R27] Haley A, Zimmerman E, Woolf S, & Evans B (2012). Neighborhood-level determinants of life expectancy in Oakland, CA. Center on Human Needs, Virginia Commonwealth University.

[R28] Halley L, Doherty MK, Megson IL, McNamara N, Gadja A, & Wei J (2013). Search for schizophrenia susceptibility variants at the HLA-DRB1 locus among a British population. Immunogenetics, 65(1), 1–7. 10.1007/s00251-012-0652-y 23053058

[R29] Joshi PK, Pirastu N, Kentistou KA, Fischer K, Hofer E, Schraut KE, Clark DW, Nutile T, Barnes CLK, Timmers PRHJ, Shen X, Gandin I, McDaid AF, Hansen TF, Gordon SD, Giulianini F, Boutin TS, Abdellaoui A, Zhao W, … Wilson JF (2017). Genome-wide meta-analysis associates HLA-DQA1/DRB1 and LPA and lifestyle factors with human longevity. Nature Communications, 8(1), 910. 10.1038/s41467-017-00934-5 PMC5715013 29030599

[R30] Jutte DP, Miller JL, & Erickson DJ (2015). Neighborhood adversity, child health, and the role for community development. Pediatrics, 135(Supplement_2), S48–S57. 10.1542/peds.2014-3549F 25733725

[R31] Kamburov A, Pentchev K, Galicka H, Wierling C, Lehrach H, & Herwig R(2011). ConsensusPathDB: Toward a more complete picture of cell biology. Nucleic Acids Research, 39(suppl_1), D712–D717. 10.1093/nar/gkq1156 21071422 PMC3013724

[R32] Kamburov A, Wierling C, Lehrach H, & Herwig R (2009). ConsensusPathDB—a database for integrating human functional interaction networks. Nucleic Acids Research, 37(suppl_1), D623–D628. 10.1093/nar/gkn698 18940869 PMC2686562

[R33] Karatsoreos IN, & McEwen BS (2013). Resilience and vulnerability: A neurobiological perspective. F1000Prime Reports, 5, 13. 10.12703/P5-13 23710327 PMC3643078

[R34] Kofler N, Corti F, Rivera-Molina F, Deng Y, Toomre D, & Simons M (2018). The rab-effector protein RABEP2 regulates endosomal trafficking to mediate vascular endothelial growth factor receptor-2 (VEGFR2)-dependent signaling. Journal of Biological Chemistry, 293(13), 4805–4817. 10.1074/jbc.M117.812172 29425100 PMC5880142

[R35] Krishnan V, Han M-H, Graham DL, Berton O, Renthal W, Russo SJ, LaPlant Q, Graham A, Lutter M, Lagace DC, Ghose S, Reister R, Tannous P, Green TA, Neve RL, Chakravarty S, Kumar A, Eisch AJ, Self DW, Lee FS, Tamminga CA, Cooper DC, Gershenfeld HK, & Nestler EJ (2007). Molecular adaptations underlying susceptibility and resistance to social defeat in brain reward regions. Cell, 131(2), 391–404. 10.1016/j.cell.2007.09.018 17956738

[R36] Kwiatkowski D, Czarny P, Toma M, Korycinska A, Sowinska K, Galecki P, Bachurska A, Bielecka-Kowalska A, Szemraj J, Maes M, & Sliwinski T (2016). Association between single-nucleotide polymorphisms of the hOGG1, NEIL1, APEX1, FEN1, LIG1, and LIG3 genes and alzheimer’s disease risk. Neuropsychobiology, 73(2), 98–107. 10.1159/000444643 27010693

[R37] Lander E, & Kruglyak L (1995). Genetic dissection of complex traits: Guidelines for interpreting and reporting linkage results. Nature Genetics, 11(3), 241–247. 10.1038/ng1195-241 7581446

[R38] Lavizzo-Mourey R (n.d.). Why health, poverty, and community development are inseparable. Federal Reserve Bank of San Francisco.

[R39] Lee Y, Choi I, Kim J, & Kim K (2016). DNA damage to human genetic disorders with neurodevelopmental defects. Journal of Genetic Medicine, 13(1), 1–13. 10.5734/JGM.2016.13.1.1

[R40] Leek JT (2020). Surrogate variable analysis (Ph.D., University of Washington). University of Washington.

[R41] Li D, Suzuki H, Liu B, Morris J, Liu J, Okazaki T, Li Y, Chang P, & Abbruzzese JL (2009). DNA repair gene polymorphisms and risk of pancreatic cancer. Clinical Cancer Research, 15(2), 740–746. 10.1158/1078-0432.CCR-08-1607 19147782 PMC2629144

[R42] Li G, Wang X, Wang X, Guan Z, Guo J, Wang F, Zhang J, Niu B, Zhang T, Wang J, & Yang J (2018). Polymorphism rs1052536 in base excision repair gene Is a risk factor in a high-risk area of neural tube defects in China. Medical Science Monitor : International Medical Journal of Experimental and Clinical Research, 24, 5015–5026. 30022792 10.12659/MSM.907492 PMC6067017

[R43] Lu AK-M, Hsieh S, Yang C-T, Wang X-Y, & Lin S-H (2023). DNA methylation signature of psychological resilience in young adults: Constructing a methylation risk score using a machine learning method. Frontiers in Genetics, 13, 1046700. 36712885 10.3389/fgene.2022.1046700 PMC9877348

[R44] Luthar SS, Cicchetti D, & Becker B (2000). The construct of resilience: A critical evaluation and guidelines for future work. Child Development, 71(3), 543–562. 10.1111/1467-8624.00164 10953923 PMC1885202

[R45] Luthar SS, Grossman EJ, & Small PJ (2015). Resilience and adversity. In Handbook of child psychology and developmental science: Socioemotional processes. (vol. 3, 7th ed. pp. 247–286). John Wiley & Sons.

[R46] Makrythanasis P, Kato M, Zaki MS, Saitsu H, Nakamura K, Santoni FA, Miyatake S, Nakashima M, Issa MY, Guipponi M, Letourneau A, Logan CV, Roberts N, Parry DA, Johnson CA, Matsumoto N, Hamamy H, Sheridan E, Kinoshita T, Antonarakis SE, & Murakami Y (2016). Pathogenic variants in PIGG cause intellectual disability with seizures and hypotonia. The American Journal of Human Genetics, 98(4), 615–626. 10.1016/j.ajhg.2016.02.007 26996948 PMC4833197

[R47] Mansell G, Gorrie-Stone TJ, Bao Y, Kumari M, Schalkwyk LS, Mill J, & Hannon E (2019). Guidance for DNA methylation studies: Statistical insights from the illumina EPIC array. BMC Genomics, 20(1), 366. 10.1186/s12864-019-5761-7 31088362 PMC6518823

[R48] Masten AS (2001). Ordinary magic: Resilience processes in development. American Psychologist, 56(3), 227–238. 10.1037/0003-066X.56.3.227 11315249

[R49] Mayes SD, Calhoun SL, Bixler EO, & Zimmerman DN (2009). IQ and neuropsychological predictors of academic achievement. Learning and Individual Differences, 19(2), 238–241. 10.1016/j.lindif.2008.09.001

[R50] McEwen BS, Gray JD, & Nasca C (2015). Recognizing resilience: Learning from the effects of stress on the brain. Neurobiology of Stress, 1, 1–11. 10.1016/j.ynstr.2014.09.001 25506601 PMC4260341

[R51] Milaniak I, Cecil CAM, Barker ED, Relton CL, Gaunt TR, McArdle W, & Jaffee SR (2017). Variation in DNA methylation of the oxytocin receptor gene predicts children’s resilience to prenatal stress. Development and Psychopathology, 29(5), 1663–1674. 10.1017/S0954579417001316 29162179

[R52] Miller O, Shakespeare-Finch J, & Bruenig D (2020). Link to external site, this link will open in a new window, link to external site, this link will open in a new window. Psychological Trauma: Theory, Research, Practice, and Policy, 12(7), 750–755. 32212777 10.1037/tra0000574

[R53] Morris TJ, Butcher LM, Feber A, Teschendorff AE, Chakravarthy AR, Wojdacz TK, & Beck S (2014). ChAMP: 450k chip analysis methylation pipeline. Bioinformatics, 30(3), 428–430. 10.1093/bioinformatics/btt684 24336642 PMC3904520

[R54] Nakamura BJ, Ebesutani C, Bernstein A, & Chorpita BF (2009). A psychometric analysis of the child behavior checklist DSM-oriented scales. Journal of Psychopathology and Behavioral Assessment, 31(3), 178–189. 10.1007/s10862-008-9119-8 PMC2914253 20700377

[R55] Nordlund J, Bäcklin CL, Wahlberg P, Busche S, Berglund EC, Eloranta M-L, Flaegstad T, Forestier E, Frost B-M, Harila-Saari A, Heyman M, Jónsson ÓG, Larsson R, Palle J, Rönnblom L, Schmiegelow K, Sinnett D, Söderhäll S, Pastinen T, Gustafsson MG, Lönnerholm G, & Syvänen A-C (2013). Genome-wide signatures of differential DNA methylation in pediatric acute lymphoblastic leukemia. Genome Biology, 14(9), r105. 10.1186/gb-2013-14-9-r105 24063430 PMC4014804

[R56] Notterman DA, & Mitchell C (2015). Epigenetics and understanding the impact of social determinants of health. Pediatric Clinics of North America, 62(5), 1227–1240. 10.1016/j.pcl.2015.05.012 26318949 PMC4555996

[R57] Osaki E, Nishina Y, Inazawa J, Copeland NG, Gilbert DJ, Jenkins NA, Ohsugi M, Tezuka T, Yoshida M, & Semba K (1999). Identification of a novel Sry-related gene and its germ cell-specific expression. Nucleic Acids Research, 27(12), 2503–2510. 10.1093/nar/27.12.2503 10359848 PMC148454

[R58] Panagopoulos I, Isaksson M, Billström R, Strömbeck B, Mitelman F, & Johansson B (2003). Fusion of the NUP98 gene and the homeobox gene HOXC13 in acute myeloid leukemia with t(11;12)(p15;q13). Genes, Chromosomes and Cancer, 36(1), 107–112. 10.1002/gcc.10139 12461755

[R59] Panter-Brick C, & Leckman JF (2013). Editorial commentary: Resilience in child development – interconnected pathways to wellbeing. Journal of Child Psychology and Psychiatry, 54(4), 333–336. 10.1111/jcpp.12057 23517424

[R60] Payton A, Van Den Boogerd E, Davidson Y, Gibbons L, Ollier W, Rabbitt P, Worthington J, Horan M, & Pendleton N (2006). Influence and interactions of cathepsin D, HLA-DRB1 and APOE on cognitive abilities in an older non-demented population. Genes, Brain and Behavior, 5(S1), 23–31. 10.1111/j.1601-183X.2006.00191.x 16417614

[R61] Peeters H, Van Gestel S, Vlietinck R, Derom C, Derom R (1998). Validation of a telephone zygosity questionnaire in twins of known zygosity. Behavior Genetics, 28(3), 159–163. 10.1023/A:1021416112215 9670591

[R62] Peter B, Wijsman EM Jr, Nato AQ, Matsushita MM, Chapman KL, Stanaway IB, Wolff J, Oda K, Gabo VB, & Raskind WH (2016). Genetic candidate variants in two multigenerational families with childhood apraxia of speech. PLOS ONE, 11(4), e0153864. 10.1371/journal.pone.0153864 27120335 PMC4847873

[R63] Rutter M (2006). Implications of resilience concepts for scientific understanding. Annals of the New York Academy of Sciences, 1094(1), 1–12. 10.1196/annals.1376.002 17347337

[R64] Simmonds MJ, & Gough SCL (2007). The HLA region and autoimmune disease: Associations and mechanisms of action. Current Genomics, 8(7), 453–465. 10.2174/138920207783591690 19412418 PMC2647156

[R65] Smith JA, Zhao W, Wang X, Ratliff SM, Mukherjee B, Kardia SLR, Liu Y, Roux AVD, & Needham BL (2017). Neighborhood characteristics influence DNA methylation of genes involved in stress response and inflammation: The multi-ethnic study of atherosclerosis. Epigenetics, 12(8), 662–673. 10.1080/15592294.2017.1341026 28678593 PMC5687339

[R66] Steptoe A, & Feldman PJ (2001). Neighborhood problems as sources of chronic stress: Development of a measure of neighborhood problems, and associations with socioeconomic status and health. Annals of Behavioral Medicine, 23(3), 177–185. 10.1207/S15324796ABM2303_5 11495218

[R67] Sun H, Kennedy PJ, & Nestler EJ (2013). Epigenetics of the depressed brain: Role of histone acetylation and methylation. Neuropsychopharmacology, 38(1), 124–137. 10.1038/npp.2012.73 22692567 PMC3521990

[R68] Szyf M, Weaver ICG, Champagne FA, Diorio J, & Meaney MJ (2005). Maternal programming of steroid receptor expression and phenotype through DNA methylation in the rat. Frontiers in Neuroendocrinology, 26(3), 139–162. 10.1016/j.yfrne.2005.10.002 16303171

[R69] Teschendorff AE, Marabita F, Lechner M, Bartlett T, Tegner J, Gomez-Cabrero D, & Beck S (2013). A beta-mixture quantile normalization method for correcting probe design bias in illumina Infinium 450 k DNA methylation data. Bioinformatics, 29(2), 189–196. 10.1093/bioinformatics/bts680 23175756 PMC3546795

[R70] Tiet QQ, Bird HR, Davies M, Hoven C, Cohen P, Jensen PS, & Goodman S (1998). Adverse life events and resilience. Journal of the American Academy of Child & Adolescent Psychiatry, 37(11), 1191–1200. 10.1097/00004583-199811000-00020 9808931

[R71] van Dongen J, Nivard MG, Willemsen G, Hottenga J-J, Helmer Q, Dolan CV, Ehli EA, Davies GE, van Iterson M, Breeze CE, Beck S, Pool Ré, van Greevenbroek MMJ, Stehouwer CDA, Kallen C. J. Hvan der, Schalkwijk CG, Wijmenga C, Zhernakova S, Tigchelaar EF, Beekman M, … Boomsma DI (2016). Genetic and environmental influences interact with age and sex in shaping the human methylome. Nature Communications, 7(1), 11115. 10.1038/ncomms11115 PMC4820961 27051996

[R72] Vanderbilt-Adriance E, & Shaw DS (2008). Conceptualizing and re-evaluating resilience across levels of risk, time, and domains of competence. Clinical Child and Family Psychology Review, 11(1), 30–58. 18379875 10.1007/s10567-008-0031-2 PMC2683037

[R73] Weaver ICG, Cervoni N, Champagne FA, D’Alessio AC, Sharma S, Seckl JR, Dymov S, Szyf M, & Meaney MJ (2004). Epigenetic programming by maternal behavior. Nature Neuroscience, 7(8), 847–854. 15220929 10.1038/nn1276

[R74] Wodtke GT, Harding DJ, & Elwert F (2011). Neighborhood effects in temporal perspective: The impact of long-term exposure to concentrated disadvantage on high school graduation. American Sociological Review, 76(5), 713–736. 10.1177/0003122411420816 22879678 PMC3413291

[R75] Yinsheng Z, Miyoshi K, Qin Y, Fujiwara Y, Yoshimura T, & Katayama T (2022). TMEM67 is required for the gating function of the transition zone that controls entry of membrane-associated proteins ARL13B and INPP5E into primary cilia. Biochemical and Biophysical Research Communications, 636, 162–169. 10.1016/j.bbrc.2022.10.078 36334440

[R76] Zeileis A (2006). Object-oriented computation of sandwich estimators. Journal of Statistical Software, 16(9), 1–16. 10.18637/jss.v016.i09

[R77] Zhang X, Moen EL, Liu C, Mu W, Gamazon ER, Delaney SM, Wing C, Godley LA, Dolan ME, & Zhang W (2014). Linking the genetic architecture of cytosine modifications with human complex traits. Human Molecular Genetics, 23(22), 5893–5905. 10.1093/hmg/ddu313 24943591 PMC4204771

[R78] Zheng SC, Breeze CE, Beck S, & Teschendorff AE (2018). Identification of differentially methylated cell types in epigenome-wide association studies. Nature Methods, 15(12), 1059–1066. 10.1038/s41592-018-0213-x 30504870 PMC6277016

[R79] Zindler T, Frieling H, Neyazi A, Bleich S, & Friedel E (2020). Simulating comBat: How batch correction can lead to the systematic introduction of false positive results in DNA methylation microarray studies. BMC Bioinformatics, 21(1), 271. 10.1186/s12859-020-03559-6 32605541 PMC7328269

