# Supplementary material for: Methylome-wide association study of multidimensional resilience
Source: Dev Psychopathol. Author manuscript; Available in PMC 2026 Mar 23. (PMC13007632; doi:10.1017/S0954579424001330)
Supplement: Vazquez supplement [file NIHMS2153103-supplement-Vazquez_supplement.docx]

SUPPLEMENTAL MATERIAL:

**Methylome-wide Association Study of Multidimensional Resilience**

**Table S1.** Pearson Correlations for Resilience Phenotypes and Cell Type Proportions

|  | Epi | Fib | B | NK | CD4T | Mono | Neutro | Blood_cell |
| --- | --- | --- | --- | --- | --- | --- | --- | --- |
| Resilience Across Domains | 0.05 | 0.02 | 0.04 | 0.10 | 0.08 | 0.02 | -0.06 | -0.05 |
| Academic Resilience | -0.01 | 0.09 | -0.07 | 0.02 | -0.01 | -0.04 | 0.01 | 0.00 |
| Social Resilience | -0.04 | 0.05 | 0.01 | -0.03 | -0.04 | 0.03 | 0.03 | 0.03 |
| Psychological Resilience | 0.08 | 0.02 | 0.02 | 0.09 | 0.04 | 0.01 | -0.08 | -0.08 |

*Note.* *p<.05; No cell type proportions were significantly associated with any four of the four resilience phenotypes.

**Table S2.** Top Ten Significant and/or Suggestive Methylation Wide Association Study Differentially Methylated Probes

| **Model** | **Probe** | **Gender** | | **Age** | | **Race/Ethnicity** | | **Zygosity** | | **Epi Cell Type** | | **Fib Cell Type** | | **NK Cell Type** | |
| --- | --- | --- | --- | --- | --- | --- | --- | --- | --- | --- | --- | --- | --- | --- | --- |
|  |  | **Beta** | **SE** | **Beta** | **SE** | **Beta** | **SE** | **Beta** | **SE** | **Beta** | **SE** | **Beta** | **SE** | **Beta** | **SE** |
| **Resilience Across Domains** | cg08862567 | -0.108 | 0.308 | **0.212*** | 0.098 | -0.156 | 0.348 | 0.662 | 0.411 | -4.486 | 2.724 | 4.778 | 19.764 | **50.725*** | 20.637 |
|  | cg15869383 | -0.192 | 0.300 | 0.167 | 0.103 | -0.107 | 0.335 | 0.393 | 0.398 | -0.674 | 2.623 | 20.216 | 19.926 | 38.483 | 20.512 |
|  | cg23044017 | -0.368 | 0.311 | **0.216*** | 0.099 | -0.109 | 0.335 | **0.805*** | 0.400 | -1.751 | 2.625 | -0.611 | 19.425 | 36.930 | 20.909 |
|  | cg02536150 | -0.053 | 0.305 | 0.169 | 0.101 | -0.112 | 0.346 | 0.735 | 0.398 | 1.056 | 2.753 | 11.653 | 19.371 | **56.623*** | 21.599 |
|  | cg24059404 | -0.169 | 0.302 | 0.196 | 0.103 | -0.195 | 0.334 | 0.587 | 0.415 | -1.933 | 2.758 | 12.363 | 19.581 | **47.085*** | 21.709 |
|  | cg24221965 | 0.008 | 0.307 | **0.218*** | 0.102 | 0.021 | 0.349 | 0.627 | 0.418 | -2.826 | 2.795 | 5.111 | 19.436 | **45.942*** | 21.387 |
|  | cg16373426 | -0.160 | 0.304 | **0.205*** | 0.101 | -0.101 | 0.342 | 0.672 | 0.406 | -3.159 | 2.998 | 8.489 | 20.792 | **49.667*** | 22.550 |
|  | cg09114799 | -0.401 | 0.306 | **0.204*** | 0.102 | -0.077 | 0.337 | 0.567 | 0.404 | -2.381 | 2.814 | 5.069 | 19.580 | **46.770*** | 21.761 |
|  | cg18056754 | -0.209 | 0.301 | 0.169 | 0.099 | 0.017 | 0.338 | 0.551 | 0.401 | -2.967 | 2.670 | 7.362 | 19.035 | **54.358*** | 21.176 |
|  | cg03078854 | -0.232 | 0.302 | **0.240*** | 0.100 | -0.009 | 0.337 | 0.547 | 0.403 | -3.774 | 2.801 | -9.326 | 20.039 | **41.959*** | 21.630 |
| **Psychological Resilience** | cg00059246 | 0.004 | 0.061 | **-0.026*** | 0.020 | **0.146*** | 0.072 | -0.014 | 0.079 | -0.698 | 0.568 | 1.466 | 4.015 | 5.005 | 4.686 |
|  | cg10674017 | 0.005 | 0.062 | **-0.036*** | 0.020 | **0.151*** | 0.070 | -0.039 | 0.082 | 0.417 | 0.548 | 7.630 | 4.025 | 3.559 | 4.626 |
| **Academic Resilience** | cg09169455 | **-0.258*** | 0.056 | 0.027 | 0.020 | 0.024 | 0.060 | 0.033 | 0.081 | -0.126 | 0.524 | 5.631 | 3.293 | 4.283 | 4.336 |
|  | cg27413290 | **-0.230*** | 0.060 | 0.038 | 0.022 | **0.139*** | 0.061 | 0.062 | 0.082 | -0.507 | 0.578 | 4.331 | 3.637 | 6.774 | 4.583 |
|  | cg23901896 | **-0.204*** | 0.059 | 0.030 | 0.021 | **0.192*** | 0.061 | 0.049 | 0.079 | -0.959 | 0.587 | 5.264 | 3.543 | 4.969 | 4.529 |
|  | cg22018084 | **-0.239*** | 0.058 | 0.017 | 0.022 | **0.128*** | 0.060 | 0.055 | 0.084 | -0.878 | 0.577 | 4.255 | 3.591 | 8.370 | 4.587 |
|  | cg03116740 | **-0.199*** | 0.060 | **0.046*** | 0.022 | 0.118 | 0.063 | 0.099 | 0.084 | 0.680 | 0.629 | 4.586 | 3.687 | 3.327 | 4.720 |
|  | cg20678377 | **-0.266*** | 0.061 | 0.035 | 0.021 | **0.122*** | 0.063 | 0.025 | 0.086 | -1.136 | 0.629 | 4.807 | 3.670 | 3.295 | 4.441 |
|  | cg09895822 | **-0.242*** | 0.060 | **0.041*** | 0.021 | **0.120*** | 0.062 | 0.063 | 0.076 | -0.158 | 0.560 | 2.123 | 3.747 | 4.386 | 4.558 |
|  | cg16444294 | **-0.266*** | 0.062 | 0.035 | 0.021 | **0.141*** | 0.062 | 0.042 | 0.080 | -0.106 | 0.534 | 4.714 | 3.630 | 4.577 | 4.451 |
|  | cg00421032 | **-0.199*** | 0.058 | 0.033 | 0.021 | **0.124*** | 0.062 | 0.077 | 0.085 | -0.495 | 0.572 | 1.627 | 3.733 | 4.153 | 4.511 |
|  | cg08857221 | **-0.199*** | 0.059 | **0.045*** | 0.021 | **0.122*** | 0.062 | 0.094 | 0.079 | 0.585 | 0.561 | 0.811 | 3.599 | 2.482 | 4.554 |
| **Social Resilience** | cg22321318 | -0.278 | 0.289 | **0.547*** | 0.103 | 0.171 | 0.322 | 0.279 | 0.398 | **10.141*** | 2.928 | -3.600 | 18.949 | -9.994 | 20.592 |
|  | cg17416722 | -0.163 | 0.287 | **0.444*** | 0.095 | 0.545 | 0.313 | -0.085 | 0.437 | 0.193 | 2.529 | -4.056 | 20.046 | -0.532 | 19.306 |
|  | cg25960393 | -0.236 | 0.280 | **0.433*** | 0.101 | 0.253 | 0.337 | 0.026 | 0.456 | -3.738 | 2.783 | -12.107 | 19.809 | 5.281 | 20.259 |
|  | cg14321269 | -0.298 | 0.287 | **0.457*** | 0.101 | -0.123 | 0.314 | 0.408 | 0.426 | 2.578 | 2.776 | -12.654 | 20.326 | -14.684 | 20.756 |
|  | cg25998860 | -0.536 | 0.295 | **0.463*** | 0.100 | 0.561 | 0.327 | **0.913*** | 0.453 | -0.086 | 2.744 | 0.211 | 21.041 | 6.062 | 20.579 |
|  | cg15559076 | -0.205 | 0.289 | **0.488*** | 0.101 | 0.233 | 0.334 | 0.289 | 0.446 | 0.096 | 2.832 | -1.530 | 20.293 | 1.718 | 20.986 |
|  | cg11070274 | -0.251 | 0.278 | **0.440*** | 0.101 | 0.273 | 0.340 | -0.001 | 0.459 | -3.296 | 2.783 | -11.904 | 20.113 | 3.582 | 20.235 |
|  | cg20424973 | -0.520 | 0.291 | **0.461*** | 0.100 | 0.533 | 0.336 | 0.439 | 0.426 | 1.323 | 2.657 | -2.834 | 19.548 | 3.309 | 20.484 |
|  | cg10985094 | -0.022 | 0.290 | **0.485*** | 0.101 | -0.159 | 0.329 | 0.461 | 0.444 | 0.888 | 2.683 | 5.493 | 19.752 | 10.023 | 20.559 |

*Note.* ‘Probe’ is the name of the CpG probe in the human reference genome hg19/GRCh37. Also shown are the signed test statistic values for regression: ‘Beta’ or regression coefficient and ‘SE’ or standard error; p<.05 is indicated by bold text and an asterick ‘*’. Only the top ten methylome-wide significant (P-value < 9 x 10^-8^) and/or suggestive (P-value < 1 x 10^-5^) MWAS DMPs are displayed for each outcome; these parameters correspond to Table 2 in the main manuscript but are presented separately merely due to space restrictions.

**Table S3.** Methylome Wide Significant and Suggestive Differentially Methylated Probes

| **Model** | **Probe** | **Chr** | **Start** | **Beta** | **Z/T-value** | **P-value** | **Gene** |
| --- | --- | --- | --- | --- | --- | --- | --- |
| Resilience Across Domains | cg08862567 | 20 | 33447234 | 80.275 | 5.161 | 2.4517E-07 | *GGT7* |
|  | cg18153279 | 12 | 112825215 | -107.720 | -5.157 | 2.5151E-07 |  |
|  | cg15869383 | 19 | 58258088 | -129.038 | -5.087 | 3.6303E-07 | *ZNF776* |
|  | cg23044017 | 19 | 36822441 | -79.445 | -5.026 | 5.0127E-07 | *LINC00665* |
|  | cg11787544 | 13 | 29257932 | 71.521 | 4.997 | 5.8137E-07 |  |
|  | cg02536150 | 10 | 17754084 | 45.363 | 4.981 | 6.3142E-07 | *STAM* |
|  | cg24059404 | 4 | 184580365 | -193.388 | -4.937 | 7.9287E-07 | *RWDD4* |
|  | cg24221965 | 15 | 81422778 | 23.580 | 4.925 | 8.4358E-07 | *C15orf26* |
|  | cg16373426 | 5 | 157079899 | 88.290 | 4.924 | 8.4989E-07 | *SOX30* |
|  | cg22500078 | 6 | 138104344 | 114.899 | 4.893 | 9.9525E-07 |  |
|  | cg09114799 | 12 | 48152514 | -242.566 | -4.881 | 1.0559E-06 | *RAPGEF3* |
|  | cg18056754 | 11 | 122955452 | 62.652 | 4.860 | 1.1719E-06 | *CLMP* |
|  | cg03078854 | 6 | 32810000 | 96.825 | 4.850 | 1.2329E-06 | *PSMB8* |
|  | cg23032249 | 6 | 69942249 | 13.053 | 4.843 | 1.2777E-06 | *BAI3* |
|  | cg01143804 | 4 | 40751844 | -112.256 | -4.824 | 1.406E-06 | *NSUN7* |
|  | cg02648847 | 1 | 167408734 | -78.393 | -4.812 | 1.4973E-06 | *CD247* |
|  | cg01316433 | 9 | 92000900 | 68.800 | 4.810 | 1.5095E-06 | *SEMA4D* |

**Table S3** continues on the following pages…

*Note.* ‘Probe’ is the name of the CpG probe in the human reference genome hg19/GRCh37, ‘Chr’ is Chromosome, ‘Start’ is the base pair location of the probe (human reference genome hg19/GRCh37), ‘Gene’ is the gene the probe is located in, and ‘Genomic Feature’ indicates if the probe is located in an intron, exon, or CpG island. Also shown are the signed test statistic values for regression: ‘Z-value’ for the dichotomous outcome of resilience across domains, ‘T-value’ for the continuous outcomes, ‘P-values’, and ‘Beta’ or regression coefficient. All methylome-wide significant (P< 9 x 10^-8^) and suggestive (P< 1 x 10^-5^) MWAS DMPs are displayed for each outcome. These are also the DMPs that were used for the enrichment analyses.

**Table S3.** (cont’d)

| Resilience Across Domains | cg04324126 | 2 | 55277571 | -116.261 | -4.808 | 1.5272E-06 | *RTN4* |
| --- | --- | --- | --- | --- | --- | --- | --- |
|  | cg17779707 | 20 | 48807326 | -207.610 | -4.805 | 1.551E-06 | *CEBPB* |
|  | cg20346695 | 2 | 203776994 | -178.423 | -4.797 | 1.6083E-06 | *CARF* |
|  | cg23013151 | 17 | 60864729 | 17.362 | 4.796 | 1.6195E-06 | *MARCH10* |
|  | cg04710629 | 2 | 191045041 | -128.412 | -4.791 | 1.662E-06 | *C2orf88* |
|  | cg00166213 | 5 | 53606451 | -122.841 | -4.787 | 1.6934E-06 | *ARL15* |
|  | cg05879499 | 5 | 6668384 | 32.962 | 4.768 | 1.8605E-06 | *SRD5A1* |
|  | cg17568035 | 17 | 27224810 | -211.214 | -4.750 | 2.0294E-06 | *DHRS13* |
|  | cg22002948 | 3 | 41235823 | 43.192 | 4.732 | 2.225E-06 | *CTNNB1* |
|  | cg19350812 | 19 | 10676863 | -41.641 | -4.701 | 2.5866E-06 | *KRI1* |
|  | cg09220171 | 11 | 98704582 | 35.471 | 4.693 | 2.6941E-06 |  |
|  | cg16123583 | 22 | 43582883 | -55.324 | -4.692 | 2.705E-06 | *TTLL12* |
|  | cg07387591 | 20 | 17208648 | 80.581 | 4.691 | 2.7222E-06 | *PCSK2* |
|  | cg03411765 | 8 | 143484815 | -32.338 | -4.683 | 2.8315E-06 |  |
|  | cg10426797 | 17 | 7169573 | 89.509 | 4.656 | 3.2266E-06 | *Y_RNA* |
|  | cg23917918 | 10 | 13385881 | 127.575 | 4.649 | 3.3293E-06 | *SEPHS1* |
|  | cg20825216 | 11 | 2274399 | 31.154 | 4.642 | 3.4475E-06 |  |
|  | cg15679813 | 22 | 45405626 | -91.393 | -4.641 | 3.4694E-06 | *PHF21B* |
|  | cg14637885 | 12 | 74416009 | 15.828 | 4.640 | 3.4768E-06 |  |
|  | cg21470464 | 7 | 95969817 | 20.612 | 4.627 | 3.7074E-06 | *RNU6-364P* |
|  | cg12372632 | 3 | 170781530 | 45.948 | 4.625 | 3.7417E-06 | *TNIK* |
|  | cg02207779 | 14 | 24701799 | -102.581 | -4.606 | 4.1025E-06 | *GMPR2* |
|  | cg21783328 | 9 | 136243031 | -195.968 | -4.606 | 4.107E-06 | *SURF4* |
|  | cg08008884 | 1 | 235377331 | 39.450 | 4.599 | 4.2451E-06 | *ARID4B* |
|  | cg08964784 | 8 | 24769500 | 15.264 | 4.598 | 4.2677E-06 | *RP11-624C23.1* |
|  | cg07917528 | 7 | 55412267 | 25.595 | 4.589 | 4.4622E-06 | *RP11-775L16.1* |
|  | cg04482075 | 16 | 1991307 | 158.995 | 4.588 | 4.4793E-06 | *MSRB1* |
|  |  |  |  |  |  |  |  |

**Table S3.** (cont’d)

| Resilience Across Domains | cg22850860 | 3 | 45902662 | 24.664 | 4.587 | 4.4944E-06 | *LZTFL1* |
| --- | --- | --- | --- | --- | --- | --- | --- |
|  | cg10214933 | 2 | 216715261 | 79.068 | 4.585 | 4.5491E-06 |  |
|  | cg24457562 | 2 | 106212100 | -23.651 | -4.581 | 4.6264E-06 |  |
|  | cg07580827 | 11 | 111943185 | 32.149 | 4.575 | 4.7552E-06 | *PIH1D2* |
|  | cg14019124 | 11 | 66611060 | -96.940 | -4.569 | 4.9014E-06 | *RCE1* |
|  | cg02761287 | 21 | 47878739 | -90.314 | -4.561 | 5.0851E-06 | *DIP2A* |
|  | cg16595404 | 10 | 12238159 | -91.628 | -4.537 | 5.7194E-06 | *CDC123* |
|  | cg17997673 | 1 | 52082396 | 5.207 | 4.530 | 5.8866E-06 | *OSBPL9* |
|  | cg18914514 | 18 | 18822122 | -80.556 | -4.529 | 5.9323E-06 | *GREB1L* |
|  | cg13680184 | 4 | 122791313 | -65.253 | -4.529 | 5.9351E-06 | *BBS7* |
|  | cg16635767 | 19 | 39574639 | -185.857 | -4.527 | 5.9777E-06 | *PAPL* |
|  | cg05734400 | 2 | 216176659 | -73.706 | -4.520 | 6.1957E-06 | *ATIC* |
|  | cg26247036 | 11 | 71814594 | -196.676 | -4.517 | 6.2642E-06 | *LRTOMT* |
|  | cg09472203 | 15 | 83378613 | -167.882 | -4.507 | 6.5823E-06 | *AP3B2* |
|  | cg14447399 | 5 | 162930289 | -67.934 | -4.505 | 6.6274E-06 | *MAT2B* |
|  | cg09555914 | 19 | 58011308 | -225.180 | -4.502 | 6.7374E-06 | *ZNF773* |
|  | cg12001456 | 7 | 157357802 | -114.115 | -4.492 | 7.0643E-06 | *PTPRN2* |
|  | cg15358052 | 14 | 69865455 | -76.804 | -4.487 | 7.2378E-06 | *SLC39A9* |
|  | cg19878597 | 14 | 53684326 | -50.856 | -4.481 | 7.4136E-06 | *AL163953.3* |
|  | cg07160800 | 5 | 177018949 | -137.125 | -4.478 | 7.5356E-06 | *TMED9* |
|  | cg09636406 | 17 | 73663133 | -100.133 | -4.477 | 7.572E-06 | *RECQL5* |
|  | cg27276059 | 6 | 75829276 | 49.473 | 4.476 | 7.5936E-06 | *COL12A1* |
|  | cg00011284 | 16 | 53469343 | -54.459 | -4.476 | 7.5958E-06 | *RBL2* |
|  | cg08159120 | 9 | 75263370 | 20.716 | 4.468 | 7.898E-06 | *TMC1* |
|  | cg14801164 | 4 | 190393518 | 17.673 | 4.466 | 7.9571E-06 | *HSP90AA4P* |
|  | cg22232107 | 8 | 124194080 | 15.622 | 4.463 | 8.0952E-06 | *FAM83A* |

**Table S3.** (cont’d)

|  | cg02981663 | 13 | 28232082 | 52.289 | 4.460 | 8.1854E-06 | *POLR1D* |
| --- | --- | --- | --- | --- | --- | --- | --- |
| Resilience Across Domains | cg22687346 | 8 | 94767371 | -174.067 | -4.456 | 8.3344E-06 | *TMEM67* |
|  | cg06996254 | 12 | 47427790 | 65.076 | 4.453 | 8.4757E-06 |  |
|  | cg14257632 | 6 | 167351815 | 73.566 | 4.450 | 8.5993E-06 | *RNASET2* |
|  | cg17610929 | 2 | 220379043 | -84.870 | -4.450 | 8.6036E-06 | *ASIC4* |
|  | cg09532899 | 15 | 97007486 | 13.178 | 4.448 | 8.6863E-06 |  |
|  | cg15720223 | 6 | 15398117 | 53.213 | 4.446 | 8.7284E-06 | *JARID2* |
|  | cg19226770 | 4 | 156921360 | 14.690 | 4.446 | 8.7477E-06 |  |
|  | cg02457826 | 20 | 30310732 | -60.153 | -4.444 | 8.8428E-06 | *BCL2L1* |
|  | cg09808985 | 14 | 89704016 | 42.398 | 4.442 | 8.9163E-06 | *FOXN3* |
|  | cg14465408 | 6 | 82980356 | 10.150 | 4.441 | 8.9379E-06 |  |
|  | cg09994724 | 11 | 123986110 | -78.918 | -4.439 | 9.056E-06 | *VWA5A* |
|  | cg23173573 | 1 | 221916860 | -146.059 | -4.438 | 9.0883E-06 | *DUSP10* |
|  | cg17689735 | 8 | 15095819 | 11.380 | 4.436 | 9.1722E-06 | *SGCZ* |
|  | cg19139691 | 2 | 86668468 | -59.727 | -4.430 | 9.426E-06 | *KDM3A* |
|  | cg22372439 | 11 | 60929244 | -48.029 | -4.428 | 9.4951E-06 | *VPS37C* |
|  | cg09163686 | 11 | 17229661 | -148.562 | -4.427 | 9.5621E-06 | *NUCB2* |
|  | cg01877778 | 7 | 157415537 | 85.967 | 4.426 | 9.6079E-06 | *PTPRN2* |
|  | cg01089060 | 10 | 97050835 | -65.715 | -4.426 | 9.6158E-06 | *PDLIM1* |
|  | cg21054179 | 12 | 49412580 | -116.318 | -4.417 | 9.9882E-06 | *PRKAG1* |
| Psychological Resilience | cg00059246 | 12 | 54337928 | 3.673 | 4.866 | 1.9571E-06 | *HOXC13* |
|  | cg10674017 | 2 | 3201975 | -15.245 | -4.689 | 4.4048E-06 | *TSSC1* |
| Academic Resilience | cg09169455 | 5 | 16843339 | -2.185 | -6.528 | 3.3989E-10 | *MYO10* |
|  | cg27413290 | 8 | 144552724 | -4.250 | -5.687 | 3.4215E-08 | *ZC3H3* |
|  | cg23901896 | 1 | 201976445 | -10.226 | -5.465 | 1.0726E-07 | *ELF3* |
|  | cg13598010 | 7 | 72838775 | -7.625 | -5.326 | 2.151E-07 |  |
|  | cg10091996 | 16 | 31548639 | -1.845 | -4.990 | 1.0988E-06 |  |

**Table S3.** (cont’d)

| Academic Resilience | cg22018084 | 2 | 69038737 | -2.543 | -4.874 | 1.8873E-06 | *ARHGAP25* |
| --- | --- | --- | --- | --- | --- | --- | --- |
|  | cg03116740 | 11 | 841334 | 3.376 | 4.799 | 2.6679E-06 | *POLR2L* |
|  | cg20678377 | 20 | 47667339 | -2.715 | -4.780 | 2.9094E-06 | *CSE1L* |
|  | cg09895822 | 14 | 105738159 | 8.444 | 4.778 | 2.947E-06 | *BRF1* |
|  | cg16444294 | 16 | 28925789 | 17.201 | 4.773 | 3.0042E-06 | *RABEP2* |
|  | cg00421032 | 4 | 22493280 | 9.058 | 4.772 | 3.0255E-06 | *GPR125* |
|  | cg08857221 | 1 | 37941360 | 4.155 | 4.694 | 4.3153E-06 | *ZC3H12A* |
|  | cg06899313 | 6 | 117394044 | -3.045 | -4.665 | 4.9154E-06 |  |
|  | cg21207593 | 17 | 33310494 | 9.232 | 4.661 | 5.0047E-06 | *LIG3* |
|  | cg11779551 | 3 | 45736062 | 4.226 | 4.626 | 5.8588E-06 | *SACM1L* |
|  | cg24374161 | 11 | 46582057 | 6.554 | 4.622 | 5.9412E-06 | *AMBRA1* |
|  | cg03706376 | 6 | 149093351 | 1.991 | 4.599 | 6.6064E-06 | *UST* |
|  | cg19548912 | 6 | 138299067 | -1.079 | -4.570 | 7.4866E-06 |  |
|  | cg14377171 | 9 | 138022130 | 3.598 | 4.560 | 7.8217E-06 |  |
|  | cg19255656 | 4 | 2816364 | 10.344 | 4.543 | 8.4385E-06 | *SH3BP2* |
|  | cg12777862 | 16 | 31548755 | -2.465 | -4.523 | 9.2109E-06 |  |
|  | cg01642827 | 7 | 925663 | 8.978 | 4.512 | 9.676E-06 | *GET4* |
| Social Resilience | cg22321318 | 7 | 157294387 | 17.100 | 5.979 | 7.2311E-09 | *AC006372.5* |
|  | cg25950792 | 22 | 26797948 | 105.089 | 5.947 | 8.5823E-09 |  |
|  | cg17416722 | 6 | 32554384 | 6.440 | 5.728 | 2.7526E-08 | *HLA-DRB1* |
|  | cg25960393 | 8 | 9106558 | 5.018 | 5.708 | 3.0643E-08 | *RP11-115J16.1* |
|  | cg14321269 | 17 | 6658197 | 17.674 | 5.546 | 7.0609E-08 | *XAF1* |
|  | cg25998860 | 5 | 126853953 | -114.782 | -5.512 | 8.3886E-08 | *PRRC1* |
|  | cg15559076 | 11 | 128109596 | 18.105 | 5.439 | 1.2196E-07 | *RP11-702B10.1* |
|  | cg11070274 | 8 | 9106609 | 5.106 | 5.278 | 2.721E-07 | *RP11-115J16.1* |
|  | cg07273698 | 2 | 46636808 | 19.462 | 5.240 | 3.2738E-07 |  |

**Table S3.** (cont’d)

| Social Resilience | cg20424973 | 2 | 3045240 | 40.116 | 5.209 | 3.8106E-07 | *LINC01250* |
| --- | --- | --- | --- | --- | --- | --- | --- |
|  | cg19815792 | 10 | 130267642 | 26.874 | 5.171 | 4.6057E-07 |  |
|  | cg10985094 | 17 | 3631481 | 23.115 | 5.064 | 7.7009E-07 | *ITGAE* |
|  | cg12738264 | 7 | 148725794 | -210.602 | -5.044 | 8.4631E-07 | *PDIA4* |
|  | cg04141477 | 10 | 71502791 | 21.169 | 5.029 | 9.0758E-07 |  |
|  | cg07694621 | 2 | 43151937 | 14.740 | 5.024 | 9.3162E-07 |  |
|  | cg15856489 | 17 | 71687902 | 16.357 | 5.021 | 9.4378E-07 |  |
|  | cg02147339 | 13 | 96632986 | 19.975 | 4.934 | 1.4241E-06 | *UGGT2* |
|  | cg06154432 | 10 | 77325337 | 12.938 | 4.924 | 1.4968E-06 | *C10orf11* |
|  | cg24147543 | 6 | 32554480 | 4.661 | 4.892 | 1.7364E-06 | *HLA-DRB1* |
|  | cg01085765 | 16 | 29139623 | 12.784 | 4.874 | 1.8835E-06 | *RP11-426C22.5* |
|  | cg14255617 | 6 | 32729117 | 13.708 | 4.843 | 2.1816E-06 | *HLA-DQB2* |
|  | cg20822540 | 1 | 9070126 | 11.551 | 4.837 | 2.2403E-06 | *SLC2A7* |
|  | cg22867288 | 6 | 57086715 | -57.579 | -4.822 | 2.3965E-06 | *RAB23* |
|  | cg04989255 | 8 | 110094904 | 19.688 | 4.807 | 2.5698E-06 |  |
|  | cg09826506 | 4 | 522635 | 41.146 | 4.796 | 2.7079E-06 | *PIGG* |
|  | cg13256398 | 10 | 64579264 | 16.940 | 4.791 | 2.7608E-06 | *EGR2* |
|  | cg09670566 | 10 | 28507576 | -95.315 | -4.782 | 2.8864E-06 | *MPP7* |
|  | cg11726507 | 14 | 101155518 | 18.507 | 4.774 | 2.9897E-06 |  |
|  | cg10506179 | 7 | 158884942 | 67.823 | 4.771 | 3.0337E-06 | *VIPR2* |
|  | cg19584551 | 10 | 24721828 | 19.546 | 4.769 | 3.0524E-06 | *KIAA1217* |
|  | cg09990723 | 2 | 242691867 | 81.620 | 4.769 | 3.0537E-06 | *D2HGDH* |
|  | cg12395012 | 8 | 11607385 | -32.546 | -4.753 | 3.2929E-06 | *GATA4* |
|  | cg24036126 | 6 | 26234818 | -79.468 | -4.706 | 4.0821E-06 | *HIST1H1D* |
|  | cg23104823 | 14 | 45553407 | -100.285 | -4.704 | 4.1139E-06 | *PRPF39* |
|  | cg01926740 | 5 | 137911360 | -104.847 | -4.689 | 4.391E-06 | *HSPA9* |

**Table S3.** (cont’d)

| Social Resilience | cg25105147 | 7 | 144474742 | 16.827 | 4.687 | 4.4442E-06 | *TPK1* |
| --- | --- | --- | --- | --- | --- | --- | --- |
|  | cg08185661 | 11 | 7273497 | -73.812 | -4.685 | 4.482E-06 | *SYT9* |
|  | cg23978866 | 2 | 47230406 | 13.203 | 4.638 | 5.5428E-06 | *TTC7A* |
|  | cg10327502 | 20 | 37570621 | 38.265 | 4.609 | 6.2832E-06 | *FAM83D* |
|  | cg20140488 | 22 | 25463865 | 12.812 | 4.601 | 6.5321E-06 | *KIAA1671* |
|  | cg05148288 | 9 | 129319931 | 77.926 | 4.590 | 6.8552E-06 |  |
|  | cg00556742 | 2 | 200820714 | -132.159 | -4.587 | 6.9569E-06 | *C2orf47* |
|  | cg25214900 | 12 | 79693301 | 26.200 | 4.574 | 7.3382E-06 | *SYT1* |
|  | cg15457276 | 19 | 4832023 | -111.973 | -4.561 | 7.8039E-06 | *TICAM1* |
|  | cg24607831 | 14 | 76975801 | 26.404 | 4.559 | 7.8588E-06 | *RP11-187O7.3* |
|  | cg02384897 | 22 | 30214218 | 24.545 | 4.545 | 8.3512E-06 | *ASCC2* |
|  | cg24945222 | 16 | 4395036 | 15.478 | 4.542 | 8.4858E-06 | *CORO7-PAM16* |
|  | cg12312265 | 10 | 72546530 | 22.293 | 4.540 | 8.5565E-06 | *TBATA* |
|  | cg10572362 | 3 | 125742863 | 38.532 | 4.530 | 8.9146E-06 | *SLC41A3* |
|  | cg14402217 | 2 | 71222107 | -157.326 | -4.530 | 8.9202E-06 | *AC007040.6* |
|  | cg10544696 | 10 | 1585344 | 11.113 | 4.526 | 9.0675E-06 | *ADARB2* |
|  | cg00695187 | 11 | 48032703 | 16.950 | 4.526 | 9.0732E-06 | *PTPRJ* |
|  | cg03384047 | 6 | 157357516 | 15.729 | 4.516 | 9.5091E-06 | *ARID1B* |
|  | cg17933911 | 1 | 59248877 | 95.814 | 4.509 | 9.7689E-06 | *JUN* |
|  | cg23847172 | 7 | 124406111 | -112.998 | -4.508 | 9.8334E-06 | *GPR37* |
|  | cg13988209 | 11 | 69683042 | 16.937 | 4.507 | 9.8622E-06 |  |
|  | cg20332503 | 7 | 143081286 | 16.673 | 4.506 | 9.9191E-06 | *ZYX* |
|  | cg10594585 | 1 | 153756108 | 21.460 | 4.506 | 9.9293E-06 |  |
|  | cg07674022 | 4 | 122854329 | 10.007 | 4.506 | 9.932E-06 | *TRPC3* |
|  | cg23123972 | 14 | 23080612 | 51.672 | 4.504 | 9.9926E-06 | *ABHD4* |
